# Size-Controlled High-Temperature Synthesis of Crystalline Niobium and Tantalum Oxide Nanoparticles: Exploring Structural Variations at Nanoscale

**DOI:** 10.1021/acs.inorgchem.5c04574

**Published:** 2025-11-17

**Authors:** Philipp Pfeifer, Souriddha Sanyal, Marko Malinovic, Andreas Göpfert, Andreas Hutzler, Huize Wang, Marc Ledendecker

**Affiliations:** a Technical University of Munich, Campus Straubing for Biotechnology and Sustainability, Sustainable Energy Materials, Schulgasse 22, 94315 Straubing, Germany; b Helmholtz Institute Erlangen-Nürnberg for Renewable Energy, 28334 Forschungszentrum Jülich GmbH, Cauerstraße 1, 91058 Erlangen, Germany

## Abstract

Niobium and tantalum oxides are highly stable materials under harsh oxidizing conditions with diverse applications in catalysis, energy storage, and optoelectronics. Niobium oxides exhibit a rich variety of polymorphs and crystal structures (e.g., T-, M-, H-Nb_2_O_5_, NbO_2_, and NbO), whose electronic properties depend strongly on their crystalline structure. Realizing these crystal structures typically requires thermal syntheses at high temperatures and different gas atmospheres, which causes particle sintering and growth, compromising nanoscale features and limiting functionality. Here, we present a synthesis route for niobium and tantalum oxide nanoparticles below 10 nm, showing unprecedented control over size and stoichiometry at temperatures up to 1100 °C. The synthesis utilizes a reverse microemulsion for the controlled synthesis of the nanoparticles, followed by silica shell encapsulation, which successfully preserves particle size while enabling access to different polymorphs. Structural characterization by HAADF-STEM and XRD confirms particle size preservation and the formation of various crystal structures through different heat treatments. For Nb_2_O_5_ polymorphs, the optical bandgap can be tuned by the crystal structure excluding size effects due to the uniform particle size. This approach yields highly crystalline nanoparticles with defined structures, providing model materials for a range of applications and potentially extendable to other material systems.

## Graphical abstract

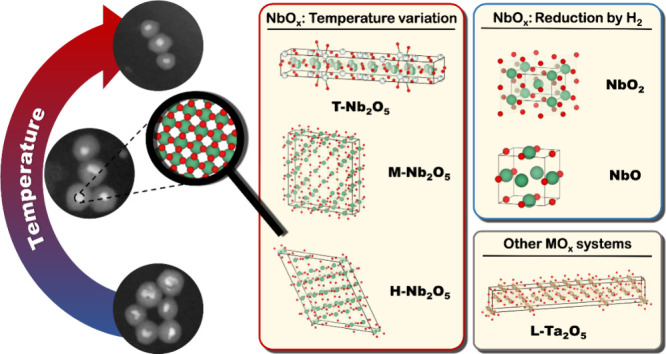

## Introduction

Valve metal oxides, particularly those based on niobium and tantalum, have emerged as versatile materials with great potential for a wide range of applications. When engineered at the nanoscale, these oxides exhibit unique properties distinct from their bulk counterparts. Tailoring their size allows for precise tuning of their electronic, optical, and catalytic properties. These properties have made valve metal oxides promising candidates in fields such as energy storage, catalysis, sensing, and electronics. Notably, tantalum oxide nanostructures have shown great promise in high-k dielectric materials and biomedical applications as well as in catalysis as a support material.^1,2^

Niobium oxides, in particular, have garnered significant attention due to their semiconductive and optical properties, as well as their structural versatility. The oxides of niobium are found in the oxidation states of II, IV, and V, each exhibiting distinct crystal structures and properties. For instance, niobium monoxide (NbO) can crystallize in two cubic structures: a rock salt structure (*Fm*3̅̅*m*) and a primitive structure (*Pm*3̅̅*m*) with Nb vacancies, both showing metallic conductivity. The latter structure also exhibits superconductive properties below 1.38 K.^3^ Niobium dioxide (NbO_2_) presents temperature-dependent polymorphism, adopting a tetragonal structure (*I*4_1_/*a*) at lower temperatures and transitioning to a conductive rutile structure (*P*4_2_/*mnm*) at higher temperatures. Niobium pentoxide (Nb_2_O_5_), on the other hand, exemplifies complex polymorphism, with multiple crystal structures that can be challenging to distinguish. To mitigate this complexity, we follow the German-based nomenclature established by Brauer^4^ and Schäfer et al.,^5^ which is widely accepted in the literature. This nomenclature is primarily based on the relative temperatures at which the respective crystal structures appear. The formation of Nb_2_O_5_ polymorphs begins with amorphous niobium pentoxide transforming into the TT-Nb_2_O_5_ modification (“low low”) at around 500 °C. This pseudohexagonal structure (*P*6/*mmm*) consists of interconnected chains of [NbO_6_] octahedra and exhibits a higher number of defects, vacancies, and unsaturated Nb=O bonds.^6^ As temperature increases, TT-Nb_2_O_5_ transforms into the more ordered T-Nb_2_O_5_ (“low”) with an orthorhombic structure (*Pbam*).^7^ At higher temperatures, intermediate modifications like M-Nb_2_O_5_ (“medium”) and B-Nb_2_O_5_ (“sheets”) are formed. Finally, at about 1000 °C H-Nb_2_O_5_ (“high”), the most thermodynamically stable niobium pentoxide polymorph with a monoclinic structure (*P*2/*m*) is formed.^3^ Unlike the lower oxides, Nb_2_O_5_ polymorphs do not exhibit metallic conductivity but instead possess bandgaps that depend strongly on both, crystal structure and morphology. For bulk materials, a direct bandgap of 3.1 to 3.4 eV is usually reported.^8–10^

The structural complexity of niobium oxides is reflected in the variety of different applications that utilize these different properties. The bandgap energies of Nb_2_O_5_ allow for the use in photocatalysis,^11–17^ as an electrode material for lithium-based batteries and supercapacitors,^18–20^ and in solar cells.^21^ Their high chemical and thermal resistance makes them suitable for application under harsh conditions. Amorphous Nb_2_O_5_ contains acid sites and can be used as a thermal catalyst.^22^ Nb_2_O_5_ and Ta_2_O_5_ are also studied in medical applications such as drug delivery, biosensing, and contrast agents.^23^ NbO_2_ could be an interesting catalyst for the CO_2_ reduction reaction with a high selectivity toward formic acid and methanol, as well as electrochemical N_2_ fixation.^24,25^

The mechanism that leads to the formation of crystalline niobium oxides depends on the precursors and synthesis technique used. To study the short-range and long-range ordering in materials, total scattering XRD measurements can be used. In nonaqueous solvothermal synthesis from NbCl_5_ in benzyl alcohol, Alling-Frederiksen et al.^26^ showed that [NbO_6_] octahedra are formed upon exchange of chloride ligands that immediately polymerize to networks of mainly corner- or edge-sharing octahedra, depending on the temperature of the solvothermal synthesis. Kjær et al.^27^ conducted a similar study of NbCl_5_ in ethanol and isopropanol. They concluded that upon heating, polymeric clusters of [NbO_6_] octahedra are formed that act as nucleation sites for further particle growth of the crystalline nanoparticles in solution. The initial clusters are best described as corner-sharing ReO_3_-type structures that transformed during particle growth to Wadsley-Roth type structures with more edge-sharing octahedra, similar to the H-Nb_2_O_5_ structure. Using niobium ethoxide as precursor in a hydrolysis reaction, Onur et al.^28^ proved that the formed amorphous niobium oxide is made up of a variety of different [NbO_6_], [NbO_7_], and [NbO_8_] polyhedral motifs. When calcinating the amorphous powder, first reflections were observed at 540 °C during heating and identified as TT-Nb_2_O_5_. Using total scattering XRD experiments, the authors followed the evolution of this phase in situ during heat treatment. The transformation from amorphous to crystalline started at around 450 °C, with the amount of edge-sharing octahedra increasing, which is attributed to more flexibility in the crystalline structure. Notably, with increasing temperature, the size of the crystalline domains increased, indicating that the crystallization at 540 °C is not completed, and crystalline and amorphous domains coexist next to each other.^28^ When using Nb or NbO_2_ as precursor for calcination, at first an amorphous Nb_2_O_5_ is formed that then crystallizes in the low temperature polymorphs TT- and T-Nb_2_O_5_ and transforms with higher temperatures to M- or B-Nb_2_O_5_, and if a high enough temperature is reached, to H-Nb_2_O_5_.^29,30^

Different methods are known for the synthesis of nanostructured NbO_*x*_ and TaO_*x*_ materials. In sol–gel and precipitation reactions in liquid media, the synthesis conditions can be changed to achieve nanostructured (15–30 nm), amorphous materials, where crystallinity is achieved in subsequent heat treatment.^1,2,28,31–35^ In hydrothermal and solvothermal methods, applying temperatures of 100–300 °C for several hours up to multiple days allows the formation of crystalline nanostructures in solution (20–80 nm).^17,32^ Complex nanostructures, such as rods with diameters ranging from 5 to 50 nm and lengths between 100 and 500 nm, as well as sheets and cones, can be fabricated.^32^ Lower oxides have not been synthesized using these techniques as a reducing agent, such as carbon or hydrogen, and sufficiently high temperatures are required.^25,34^ Despite the promising attributes of niobium and tantalum oxides, accessing certain polymorphs on the nanoscale has been historically challenging due to the high-temperature treatments required. As temperatures increase, nanoparticles are prone to sintering and coalescence, resulting in the loss of their nanoscale features and size-dependent properties. This limitation has significantly impeded the full exploitation of the diverse Nb_*x*_O_*y*_ polymorphs in nanoscale applications. In Table S1, we give an overview of the size and synthesis of amorphous and crystalline nanoparticles of niobium and tantalum oxides in the literature and compare them with materials from this work.

To address this longstanding issue, we present an innovative approach that involves incorporating niobium and tantalum oxide nanoparticles into a silica matrix. This method enables high-temperature treatments while maintaining the nanoscale dimensions of the particles, effectively circumventing the sintering problem. By isolating the nanoparticles within the silica framework, we can access the full spectrum of Nb_*x*_O_*y*_ crystal structures without compromising the nanoscale nature of the material. This approach offers several key advantages: (1) it allows for the synthesis of high-temperature Nb_*x*_O_*y*_ polymorphs and crystalline Ta_2_O_5_ that were previously inaccessible at the nanoscale, expanding the range of potential applications; (2) the preserved nanoscale features ensure retention of the high surface area and unique physicochemical properties associated with nanoparticles; (3) the silica matrix provides additional stability and protection to the nanoparticles, potentially enhancing their longevity and performance in various applications; (4) this method opens up new possibilities for studying the intrinsic properties of different niobium and tantalum oxide materials at the nanoscale, contributing to a deeper understanding of structure–property relationships in these materials.

## Experimental Section

### Chemicals and Materials

Acetone (technical grade, Carl Roth), ammonia solution (28–30%, Merck KGaA), Brij L4 (*M*_n_ ∼ 362, Sigma-Aldrich), *n*-heptane (99.5%, Thermo Scientific), *n*-heptane, dry (99+%, Thermo Scientific), niobium­(V) ethoxide (99.99%, Fisher Scientific), tantalum­(V) ethoxide (99.999%, Fisher Scientific), tetraethyl orthosilicate (98%, Acros Organics) and water (deionized) were purchased and used as received.

Hydrogen, nitrogen, and oxygen gas cylinders were purchased from Westfalen and Linde (5.0).

### Synthesis of Metal Oxide@SiO_2_ Nanoparticles (MO_*x*_@SiO_2_ NPs)

For a typical synthesis, 88 mL of *n*-heptane are mixed with 11.5 mL Brij L4 in a round-bottom flask. Then 2.5 mL of deionized water and 1.25 mL of concentrated ammonia solution are added and kept stirring for at least 30 min to generate the reverse microemulsion. Parallelly, 12 mL of dry *n*-heptane are added to a round-bottom Schlenk flask. Through a rubber septum, 0.964 mmol of metal alkoxide precursor are added and mixed with the solvent. The whole mixture is added dropwise over a time of 5 min to the reverse microemulsion using a syringe and stirred at room temperature for 2 h. Using ammonia solution, the pH is adjusted to 10–11 before 7.5 mL of tetraethyl orthosilicate are added, and stirring is continued overnight (usually 15 h). The reaction is stopped by adding 75 mL methanol and stirring for some minutes. The particles are allowed to settle before the supernatant liquid is decanted. They are then washed and separated using centrifugation twice with methanol and a third time with acetone to ensure the removal of any remaining surfactant molecules. The nanoparticles are collected and dried in a vacuum oven at 60 °C for 1 h.

### Thermal Annealing Studies

About 100 mg of the MO_*x*_@SiO_2_ NPs are charged in an alumina crucible and placed in the isothermal zone of a tubular furnace (Nabertherm RHTC 80-230/16). For oxidation experiments, the gas stream is set to 10 L·h^–1^ of 80% N_2_ and 20% O_2_. The temperature is increased by 2 K·min^–1^ to the desired dwelling temperature and held for 3 h. Materials from this study are named “MO_*x*_@SiO_2_-T °C” with M being Nb or Ta and T being the dwelling temperature.

For studies in reductive atmosphere, the temperature is increased with 2 K·min^–1^ to 900 °C and held there for 4 h. The gas stream is set to 10 L·h^–1^ and varied in composition between 100% N_2_ and 0% H_2_ to 20% N_2_ and 80% H_2_. Materials from this study are named “NbO_*x*_@SiO_2_-X % H_2_” with X being the volume fraction of hydrogen in the gas stream.

### Material Characterization

The size, morphology and elemental distribution of the synthesized particles were analyzed using a JEOL JEM-1400Plus transmission electron microscope (120 kV, LaB_6_), a JEM2100F (JEOL) microscope (200 kV, ZnO/W(100)-emitter) and a Thermo Fisher Scientific Talos F200i (S)­TEM (200 kV, X-FEG). High-angle annular dark field scanning transmission electron microscopy (HAADF-STEM) and energy dispersive X-ray spectroscopy (STEM-EDXS) was performed with the latter at a beam current of 40 pA and a convergence angle of 10.5 mrad. The samples were prepared by dispersion of the investigated material in ethanol with ultrasonication, followed by drop-casting 30–50 μL of the dispersion on lacey carbon-supported metal grids.

Ex situ powder X-ray diffractometry (XRD) measurements were performed in a Rigaku Miniflex 600 using Cu Kα radiation between 5 and 90° 2θ with 0.01° 2θ increment and a scan rate of 1° 2θ·min^–1^. Rietveld Refinement was performed using the *Profex* software.^36^ Experimental details like refinement parameters, crystal structure data and crystallite size determination are explained in more detail in the Supporting Information. For in situ XRD measurements a Reactor X attachment in a Rigaku SmartLab SE was used. The measurements were conducted with Cu Kα radiation between 20 and 60° 2θ with 0.05° 2θ step size and a scan rate of 5° 2θ·min^–1^. To mimic the heat treatment in the tubular furnace, the temperature was increased by 2 K·min^–1^ to 1000 °C and kept there for 3 h. After the dwelling time, the reactor was cooled down by 5 K·min^–1^ to 500 °C and from there by 2 K·min^–1^ to room temperature. During the whole temperature program, XRD diffractograms were continuously recorded, about every 10 min.

Thermogravimetric analysis and differential scanning calorimetry (TGA-DSC) were carried out using 70 μL alumina crucibles in a Linseis STA PT 1600. The atmosphere was set to 20 mL·min^–1^ of synthetic air, and the temperature increased to 1000 °C using a 5 K·min^–1^ ramp. The measurement was corrected using a previously recorded zero curve.

UV–vis diffuse total reflectance measurements were done with a Shimadzu UV-2600i UV–vis Spectrophotometer with an integrating sphere. The samples and the BaSO_4_ reference were placed between two quartz glass sheets and measured between 500 and 250 nm. For more details, see the Supporting Information.

### Caution!

Niobium­(V) and tantalum­(V) ethoxides are moisture-sensitive and hydrolyze exothermically upon contact with water. Ammonia solution is corrosive, and organic solvents such as *n*-heptane, methanol, and acetone are highly flammable; they must be handled in a well-ventilated fume hood away from ignition sources. Hydrogen is a GHS Flammable Gas, Category 1. All thermal annealing studies were conducted in a tubular furnace within a ventilated enclosure connected to an exhaust system with leak-tested lines. The gas system layout must ensure that the simultaneous supply of oxygen and hydrogen gases is prevented under any operating conditions. Nanoparticles are considered potentially hazardous; all powders were handled in a fume hood with gloves and protective clothing to reduce airborne exposure.

## Results and Discussion

Building upon this approach, we employed a reverse microemulsion (RME) synthesis method to fabricate silica-encapsulated niobium oxide and tantalum oxide nanoparticles. The RME system consisted of *n*-heptane as the organic phase, a nonionic surfactant, and a mixture of water and ammonia solution as the aqueous phase. To initiate the synthesis, niobium­(V) ethoxide or tantalum­(V) ethoxide, respectively, serving as the precursor, was first solvated in *n*-heptane added to the microemulsion over a span of 5 min allowing for the controlled formation of hydrous niobium oxide intermediates in the water pools of the RME.

From literature, it is known that a niobium oxyhydroxide, or also called hydrous niobium oxide, is formed during the hydrolysis reaction.^28,37^ To ensure a complete reaction, the stirring at room temperature was continued for 2 h before tetraethyl orthosilicate (TEOS), the precursor for the silica shells, was added. The core–shell nanoparticles were collected, washed with acetone for removal of excess surfactant, followed by drying. The synthesized powder was divided into portions and subjected to different heat treatments at various temperatures and gas atmospheres.

To elucidate the thermal stability and morphological evolution of the silica-encapsulated niobium oxide nanoparticles, we conducted a comprehensive investigation into the effects of calcination temperature. Samples were calcined in a tubular furnace under controlled gas flow of 10 L·h^–1^ synthetic air for 3 h of isothermal period at different temperatures ranging from 800 to 1000 °C. The resulting materials were characterized using ex situ high-angle annular dark-field scanning transmission electron microscopy (HAADF-STEM), complemented by thermogravimetric analysis and differential scanning calorimetry (TGA-DSC) to probe the thermal behavior of the synthesized powder. Figure 1 shows the ex situ HAADF-STEM images of the samples calcinated at 800 °C, 900 °C, and 1000 °C. The average diameter of the NbO_*x*_ core encapsulated within the SiO_2_ shell remains virtually constant, exhibiting only slight variations from 9.3 ± 1.5 nm to 9.7 ± 1.7 nm across the entire temperature range. Notably, our earlier work with iridium oxide core particles demonstrated size retention up to 800 °C.^38^ The current study pushes this boundary further, showcasing the ability of silica encapsulation to prevent sintering of niobium oxide nanoparticles even at a temperature of 1000 °C. For tantalum oxide, thermal stability is extended even further, with size retention observed up to 1100 °C, as discussed later in this work.

**Figure 1:**
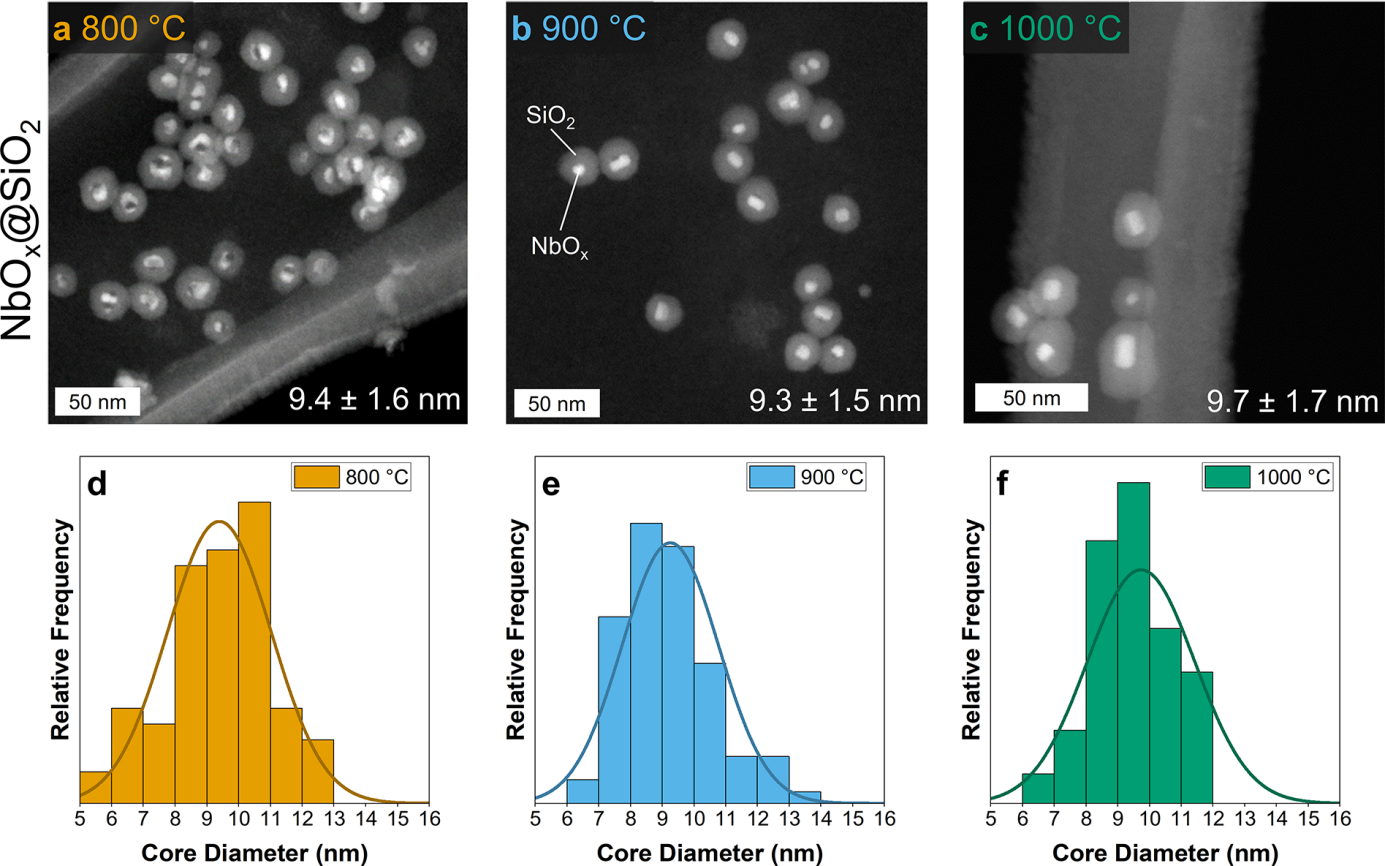
HAADF-STEM images of NbO_*x*_@SiO_2_ nanoparticles after different calcination temperatures (a–c) and average size distribution of NbO_*x*_ cores (d–f).

To elucidate the thermal behavior and phase evolution of the synthesized niobium oxide particles, TGA-DSC was performed from ambient temperature to 1000 °C (Figure S1). The TGA profile revealed a two-step weight loss process totaling 16 wt %. The initial 10 wt % loss (RT-300 °C) with only weak DSC signals corresponds to the removal of physisorbed water and the dihydroxylation of surface hydroxyl groups. Between 270 and 310 °C, a weight drop of 1 wt % accompanied by a sharp exothermic peak suggests an exothermic decomposition reaction or the burning of an organic residue. The subsequent 5 wt % loss (300–700 °C) is attributed to the further condensation of Nb–O networks and the release of water, leading to the formation of more ordered crystalline phases from the initially amorphous networks. After reaching a constant mass, the main DSC feature is a wide exothermic event between 650 and 850 °C, which could be attributed to the crystallization of the niobium oxide core.

During the hydrolyzation reaction of niobium­(V) ethoxide in the reverse microemulsion, a niobium oxyhydroxide intermediate is formed.^28,37^ Upon thermal treatment in an oxidizing atmosphere, this intermediate undergoes condensation reactions, resulting in the formation of Nb_2_O_5_ with the concomitant release of water. A similar process occurs simultaneously in the silica networks that encompass the niobium oxide particles.

Following the STEM analysis, we systematically investigated the influence of different synthesis parameters on the formation of crystalline niobium oxide structures. Leveraging the polymorphic nature of niobium oxides and their diverse stoichiometries, we employed precisely controlled thermal treatments in a tubular furnace to generate diverse structures. Our experimental design focused on two critical variables: (1) temperature variation in synthetic air atmosphere to realize different polymorphs of Nb_2_O_5_. (2) At a temperature of 900 °C, the composition of the gas stream was systematically varied introducing various ratios of H_2_ and N_2_ to explore the reduction of niobium and access oxides with different oxidation states. Ex situ X-ray diffraction (XRD) analysis was performed to identify the resulting crystal phases. For samples exhibiting well-defined crystallinity, Rietveld refinement was conducted to determine crystallite sizes and quantify phase compositions and crystallinity. The XRD patterns from the temperature-dependent study are presented in Figure 2, while those from the reductive study are shown in Figure 4.

**Figure 2:**
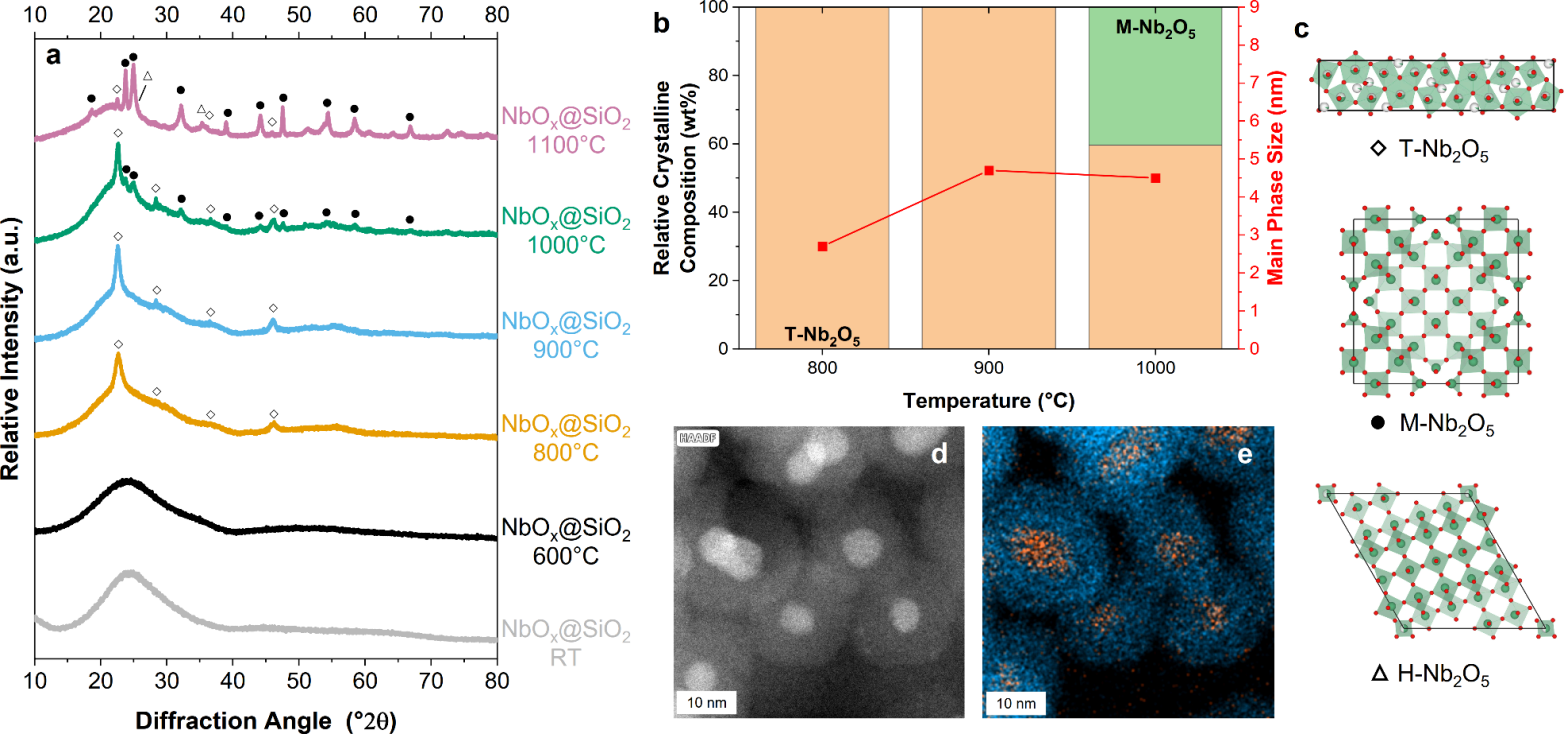
XRD diffraction patterns of NbO_*x*_@SiO_2_ samples after heat treatments in synthetic air at different temperatures (a), crystal phase composition and crystallite size of the dominant crystalline phase determined through Rietveld refinement (b), and unit cells of T-Nb_2_O_5_,^7^ M-Nb_2_O_5_,^39^ and H-Nb_2_O_5_ ^40^ (c). Additional HAADF-STEM image (d) of sample NbO_*x*_@SiO_2_-900 °C and EDX elemental map showing the distribution of niobium (orange) and silicon (blue) (e).

After calcination in synthetic air, the samples were characterized using X-ray diffraction (Figure 2a) and Rietveld refinement (Table S3 and Figure S3) of the crystalline specimen. Additionally, to prove the core–shell nature of the particles and see for crystallinity in the sample, the NbO_*x*_@SiO_2_-900 °C sample was studied using high-resolution STEM (HR-STEM) and energy dispersive X-ray (EDX) mapping (Figure 2d–e).

The as-synthesized powder does not show any crystalline reflections, which indicates the amorphous nature of a hydrous niobium oxide in SiO_2_. Likewise, the sample calcined at 600 °C shows no crystalline reflections. The broad peak at 22.4° 2θ, which is also present in the crystalline samples, can be attributed to amorphous silica. Crystalline reflections are first observed in the diffraction pattern after calcination at 800 °C. In this sample, and after calcination at 900 °C, only T-Nb_2_O_5_ is present. The HR-STEM results (Figure S11) and EDX mapping (Figure 2e) additionally reveal a well-defined core–shell morphology and indicate the crystalline nature of the NbO_*x*_ core. EDX maps of individual elements are shown in Figure S9. Lattice-resolved imaging (Figure S11) shows visible grains with an interplanar spacing of 3.87 Å in the Fourier space, corresponding to the (001) plane of T-Nb_2_O_5_ (3.93 Å).^7^ Also, in the sample calcined at 1000 °C, T-Nb_2_O_5_ is still the primary crystalline phase with 66.6%, as derived from Rietveld refinement. According to literature, the onset of Nb_2_O_5_ crystallization is reported to take place at lower temperatures between 450 to 600 °C.^5,28,29,32,41^ For mesoporous metal oxide systems, including niobium, tantalum, and niobium tantalum mixed oxides, Kondo and Domen^41^ reported that the presence of small amounts of silica in the mesopores led to an increase in the crystallization temperature. The authors attributed this observation to the decreased mobility of metal-oxide chains hindering their crystallization.^41^ A similar trend is observed in our system, where nanoparticles are encapsulated in a silica shell.

Furthermore, it is reported that TT-Nb_2_O_5_ transforms to the more ordered T-Nb_2_O_5_ at temperatures of 600 to 800 °C.^5,29,32^ However, we do not observe any reflections of TT-Nb_2_O_5_. After calcination at 1000 and 1100 °C, M-Nb_2_O_5_ is present as a minor crystalline phase (33.4% and 44.4%). After calcination at 1100 °C, H–Nb_2_O_5_ becomes the primary phase with 53.8%. Looking at the crystallite size of the major phases, there is an increase of T-Nb_2_O_5_ crystallite size from 3.76 nm at 800 °C to 4.6 nm at 1000 °C. This is in line with the STEM particle size analysis, as the crystallite size stays below the particle size. A major increase in the crystallite size is observed for NbO_*x*_@SiO_2_-1100 °C (c.f. Table S4) with 40.8 nm for M-Nb_2_O_5_, prompting further investigation using TEM imaging. The TEM images (Figure S13) revealed that at 1100 °C, the silica shells start to sinter and coalesce. This sintering process affects the niobium oxide core particles in two distinct ways: some core particles remain unaffected, maintaining their original size, while others form larger aggregates, reaching sizes up to 50 nm. These TEM observations corroborate with the Rietveld refinement results, explaining the substantial increase in crystallite size at this elevated temperature. The sintering of silica shells at high temperatures is consistent with known behavior of silica nanoparticles, as studies on silica aerogels have shown that thermal treatments can significantly affect particle morphology and surface area.^42^

To achieve improved temperature resolution during the crystallization of T- and M-Nb_2_O_5_, additional in situ XRD measurements were performed under conditions that replicate the thermal profile of the tubular furnace. The resulting diffraction patterns, recorded during the heat treatment, are presented in Figure 3 as a two-dimensional contour plot, illustrating diffraction intensity as a function of temperature and diffraction angle.

**Figure 3:**
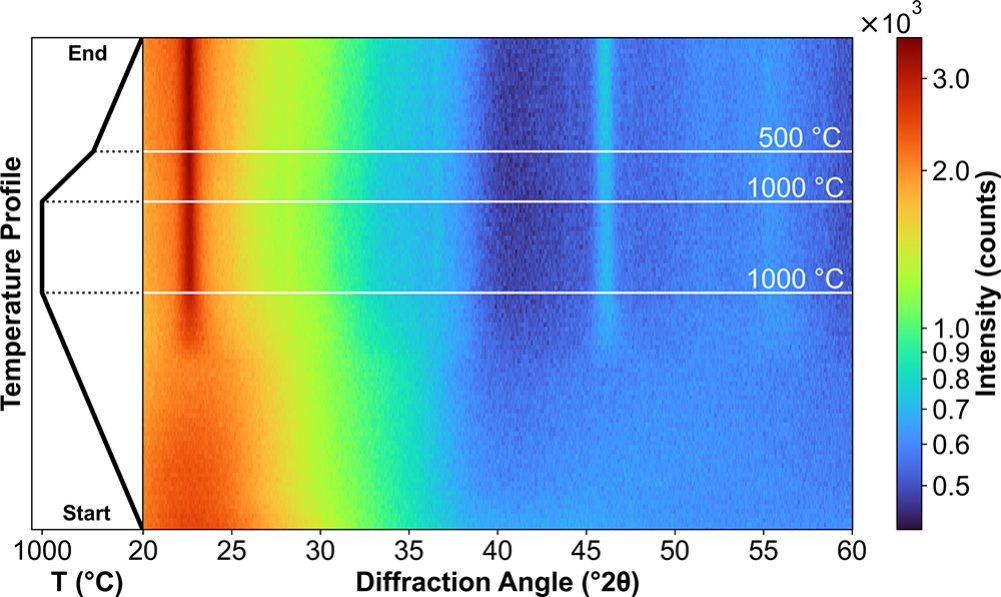
Contour plot of diffraction patterns obtained during in situ XRD measurements, while following the shown temperature profile in synthetic air. From room temperature to 1000 °C, the temperature was increased at 2 K/min and kept constant for 3 h. To mimic the cooling behavior of the tubular furnace, the temperature is then decreased with 5 K/min to 500 °C and then with 2 K/min back to room temperature. XRD diffractograms were constantly recorded with a scan rate of 5° 2θ/min. The color bar shows the recorded intensity on a logarithmic scale for better contrast.

As in the ex situ XRD measurements, the patterns are dominated by the wide amorphous peak between 20° 2θ and 30° 2θ. From 200 °C onward, the center of the amorphous peaks shifts slightly to smaller diffraction angles. Between 750 and 800 °C, the first crystalline diffraction peaks begin to emerge. Notable peaks appear at around 22° 2θ, 46° 2θ, and a broad peak centered around 35° 2θ, which can be assigned to T-Nb_2_O_5_. During the isothermal holding at 1000 °C, additional peaks of this phase become visible at 27.5° 2θ and 36.5° 2θ.

Between 950 and 1000 °C, a second phase transition is hinted at by the appearance of a broad peak at 55° 2θ. During the isothermal holding period, weak reflections appear around 45° 2θ, which are also indicative of the presence of the monoclinic M-Nb_2_O_5_ phase. However, these peaks are less pronounced compared to those observed in ex situ measurements. Nevertheless, the data suggest that the T-phase transforms into the M-phase at temperatures shortly below or around 1000 °C.

The diffraction patterns recorded at 1000 °C remain unchanged throughout the cooling phase, indicating that the final crystalline phase is established during the isothermal holding period rather than during cool-down. This observation underscores the critical role of thermal dwell time at high temperature in governing phase formation and stability. Consequently, it substantiates the rationale behind employing distinct isothermal holding temperatures to deliberately tailor the phase composition and material properties.

In addition to our temperature variation study, we conducted a comprehensive investigation into the effects of reducing atmospheres at a constant temperature of 900 °C using ex situ XRD (Figure 4a) and Rietveld refinement. The detailed results are provided in Table S5 and the fitted diffraction patterns are included in Figure S4. To exclude the influence of oxygen, the oven tube was evacuated and refilled with the desired gas composition before proceeding in continuous gas flow.

**Figure 4:**
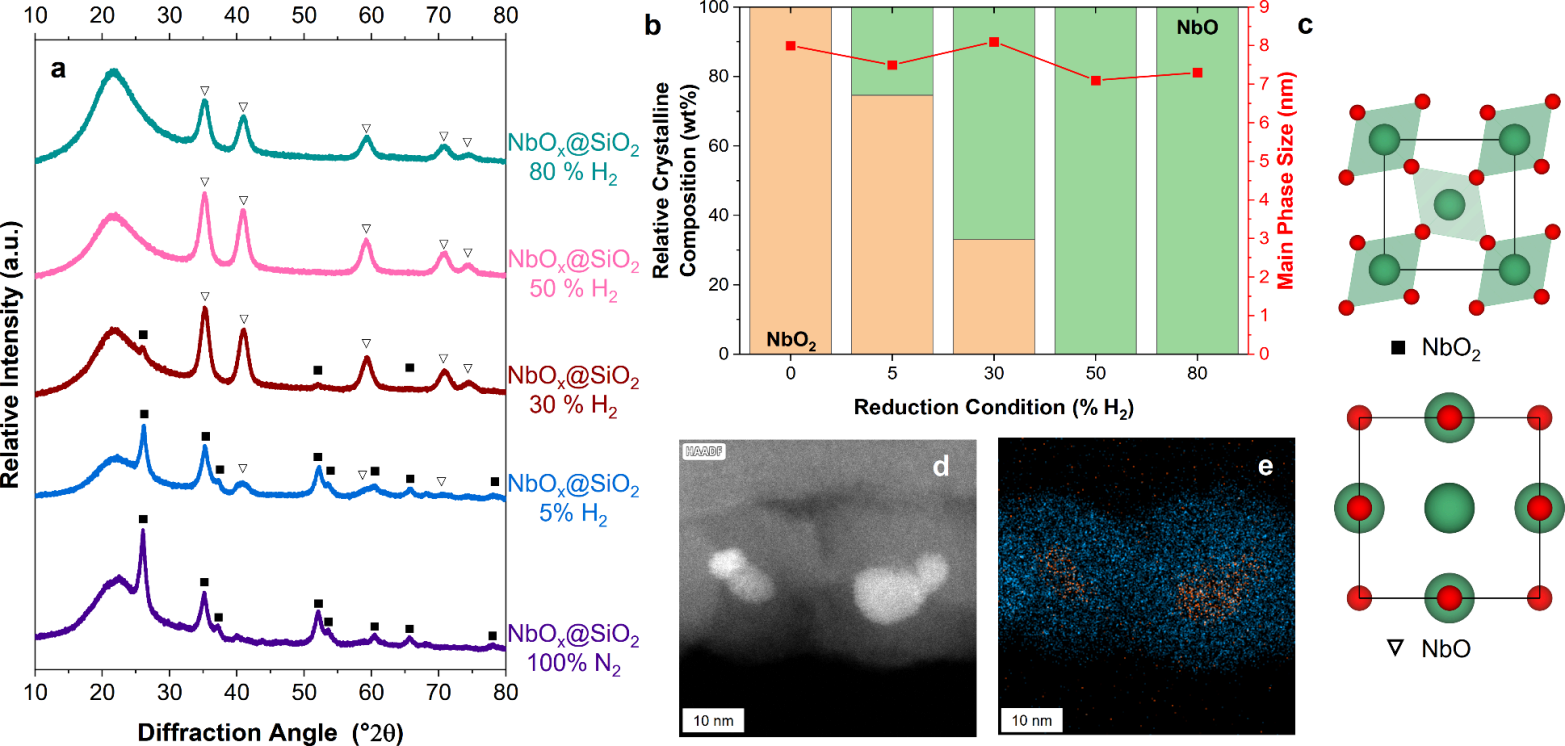
XRD diffraction patterns of NbO_*x*_@SiO_2_ samples after different heat treatments at 900 °C with different H_2_ concentrations in N_2_ (a), crystal phase composition and crystallite size of the dominant crystalline phase determined through Rietveld refinement (b), and unit cells of NbO^4^ and NbO_2_ ^43^ (c). Additional HAADF-STEM image (d) of sample NbO_*x*_@SiO_2_-50% H_2_ and EDX elemental map showing the distribution of niobium (orange) and silicon (blue) (e).

The crystallite sizes of all major phases remain below 10 nm across all reductive treatments. Additionally, all samples in this study exhibit distinct crystalline reflections. Depending on the conditions, the rutile-type high-temperature modifications of niobium dioxide and niobium monoxide can be found. NbO_2_ is observed following heat treatment in an inert atmosphere. The reduction of niobium from its initial V oxidation state in the precursor was systematically investigated through a series of controlled hydrogen treatments. Upon introduction of H_2_, crystalline reflections of NbO start to emerge in the XRD diffraction patterns. In an atmosphere of 5% H_2_ and 95% N_2_, NbO_2_ forms the major phase with 74.6%, while NbO is the minor phase with 25.4%. After reduction with 30% H_2_, NbO becomes the major crystalline phase and is the sole present phase after reduction with 50% or 80% H_2_. From XRD and Rietveld refinement, there is no qualitative difference between the samples NbO_*x*_@SiO_2_-50% H_2_ and NbO_*x*_@SiO_2_-80% H_2_, which means that for the formation of pure NbO nanoparticles, 50% hydrogen atmosphere seems to be sufficient. This finding represents a significant advancement in the synthesis of niobium monoxide nanostructures. Conventional methods for NbO production typically involve the high-temperature reaction of equimolar amounts of NbO_2_ and Nb metal, resulting in larger structures, or employ nonscalable gas-phase ion beam techniques.^44–46^ Our heat treatment method demonstrates the production of NbO via the direct reduction of hydrous niobium oxide encapsulated within SiO_2_.

To confirm the XRD results, NbO_*x*_@SiO_2_-50% H_2_ was analyzed additionally using HR-STEM imaging (Figure S12) and EDX mapping (Figure 4e). The HAADF-STEM images reveal well-defined core–shell structures, uniformly sized below 10 nm, with the NbO_*x*_ core exhibiting a crystalline nature. The measured lattice spacing of 1.61 Å corresponds to the (220) plane of NbO (1.56 Å) (Figure S12), confirming XRD and Rietveld refinement results. The HAADF-STEM and EDX elemental mapping further verifies the core–shell architecture, demonstrating a clear distinction between the Nb-rich core and the surrounding SiO_2_ matrix. This finding shows, that the silica shell can also keep the NbO_*x*_ cores from sintering in reductive conditions.

As a control experiment, we repeated the heat treatments in an inert N_2_ atmosphere and in a 50% H_2_ atmosphere using NbO_*x*_ nanoparticles without a silica shell and analyzed them for composition and crystallite size using Rietveld refinement (Figure S6 and Table S6). Interestingly, the phase composition deviates from the experiment where silica is present. In the inert atmosphere, the XRD analysis reveals T-Nb_2_O_5_ as the main phase with a crystallite size of 77.0 nm, while in a 50% H_2_ atmosphere, the crystal phase consists of 52.2% NbO_2_ (98.3 nm) and 47.8% NbO (39.87 nm). These results point out that the silica shell not only plays a role in containing the particle growth but in an oxygen-deficient or reducing environment, leads to the reduction of niobium ions by acting as an oxygen sink. According to Ellingham diagrams, which display the relative stability of oxides, SiO_2_ is more thermodynamically stable than the niobium oxides. As a result, SiO_2_ enhances oxygen uptake from the core–shell system, promoting the reduction of niobium oxides.^47,48^

While NbO and NbO_2_ are metallic conductors and potential support materials in thermo- and electrocatalysis, Nb_2_O_5_ polymorphs are semiconductors. To further elucidate the electronic properties of these materials and their dependence on synthesis conditions, we conducted a systematic investigation of the optical bandgaps of Nb_2_O_5_-based materials. The optical bandgaps of Nb_2_O_5_-based materials prepared via heat treatment in synthetic air were systematically investigated by measuring the diffuse reflectance of the samples with BaSO_4_ as the standard reference material and using the Tauc plot method as described by Makuła et al.^49^ Assuming direct electronic transitions, the bandgap energies were determined for samples heat-treated at various temperatures. The results of the Tauc plot method (Figure S16) are shown in Table 1.

**Table 1:** Determined Optical Bandgap of Different NbO_*x*_@SiO_2_ Samples after Calcination in Synthetic Air

sample	bandgap/eV
NbO_*x*_@SiO_2_-RT	4.33
NbO_*x*_@SiO_2_-400 °C	4.19
NbO_*x*_@SiO_2_-600 °C	4.14
NbO_*x*_@SiO_2_-800 °C	4.20
NbO_*x*_@SiO_2_-900 °C	4.25
NbO_*x*_@SiO_2_-1000 °C	4.12
NbO_*x*_@SiO_2_-1100 °C	4.09

The uncalcined sample exhibited the highest bandgap energy of 4.33 eV. For heat treatment temperatures between 400 and 900 °C, the bandgap energies remained relatively consistent, ranging from 4.19 to 4.25 eV. A notable decrease in bandgap energy was observed for samples calcined at temperatures ≥1000 °C, with the sample heat-treated at 1100 °C displaying the lowest bandgap of the measured samples of 4.09 eV.

These findings are consistent with literature reports on Nb_2_O_5_ materials.^8,9,50^ While bulk Nb_2_O_5_ crystals typically exhibit a bandgap of 3.4 eV, quantum size effects can significantly influence the electronic properties. Brayner and Bozon-Verduraz^8^ previously demonstrated a blue-shift of the bandgap from 3.4 to 4.2 eV when the particle size of a Nb_2_O_5_ sol decreased from 40 nm to an average of 4.5 nm, corroborating our experimental observations.

The gradual reduction in bandgap energy, particularly after calcination at higher temperatures, can be attributed to the emergence of the M- and H-Nb_2_O_5_ crystalline phases, present in the NbO_*x*_@SiO_2_-1000 °C and NbO_*x*_@SiO_2_-1100 °C samples. Literature suggests that both crystalline phases possess smaller bandgap energies than the low-temperature polymorphs.^8^

Our results demonstrate that heat treatment up to 1000 °C enables tuning of the bandgap energy, primarily influenced by changes in the crystalline phase. Notably, particle size effects appear minimal within this temperature range.

In the preceding sections, niobium oxide was utilized as a model system to elucidate key concepts of the crystallization behavior of silica-encapsulated metal oxide nanoparticles at high temperatures. This methodology can be extended to synthesize crystalline nanoparticles of other transition metal oxides such as tantalum oxide. Due to the similar reactivity of tantalum and niobium, the synthesis protocol allows for direct substitution of niobium ethoxide with an equimolar amount of tantalum ethoxide. Hydrolysis of the precursor followed by implementation of the silica precursor yields amorphous TaO_*x*_@SiO_2_ nanoparticles, which subsequently transform to crystalline orthorhombic L-Ta_2_O_5_ (*Pmm*2) when heat treatment in synthetic air is applied.^31^

To investigate the nanoparticle morphology and crystalline properties, the previously employed methodology for the niobium oxides is applied to the tantalum oxide system, utilizing STEM analysis (Figure 5a–d) for particle size and XRD (Figure 5e) and Rietveld refinement (Table S6 and Figure S5) to access the crystalline properties. Additional EDX mapping is also available in Figure S15.

**Figure 5:**
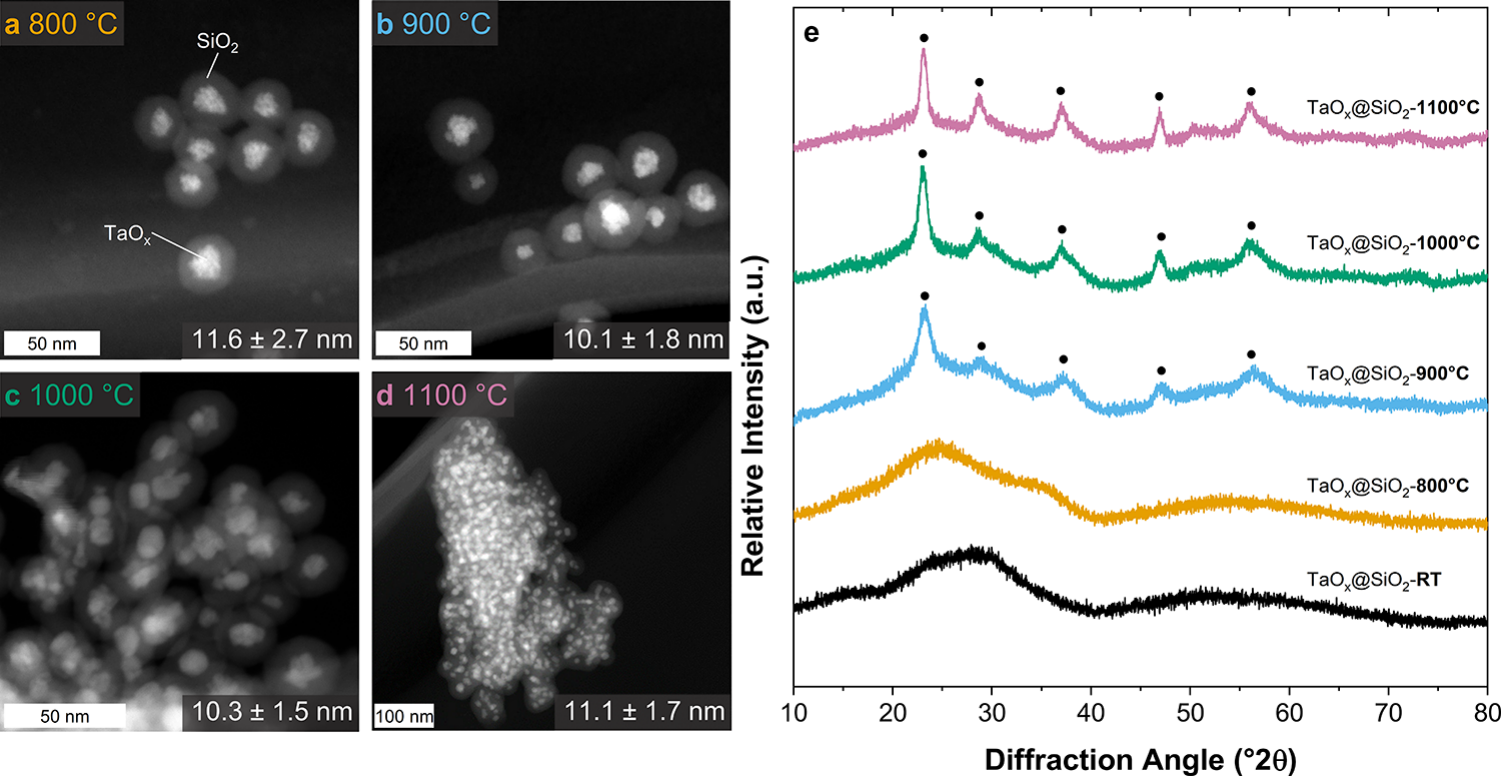
HAADF-STEM image of TaO_*x*_@SiO_2_ calcinated at 800 °C (a), 900 °C (b), 1000 °C (c), and 1100 °C (d) and XRD diffraction patterns taken after calcination at different holding temperatures (e).

The STEM results demonstrate that the core–shell structure remains intact until 1000 °C. At 1100 °C, the silica shells start to sinter, but the Ta_2_O_5_ cores remain separated. Interestingly and conversely to niobium oxide, the particle size was kept constant up to 1100 °C in the range of about 10–12 nm. Crystalline reflections of tantalum oxide become observable at temperatures of 900 °C and above. Notably, this crystallization temperature for TaO_*x*_@SiO_2_ particles exceeds that reported in the literature.^2,31,41^ As for Nb_2_O_5_, this can be attributed to the encapsulation in SiO_2_.^41^ In contrast to the variety in Nb_2_O_5_ polymorphs, only one crystalline phase is reported in this temperature range. Rietveld refinement analysis reveals that crystallite sizes increase from 3.03 nm at 900 °C to 6.33 nm at 1100 °C, remaining below the particle size measured in STEM.

In comparison with the niobium sample at the same temperature, Ta_2_O_5_@SiO_2_-1100 °C does not show increased particle size in either STEM or Rietveld refinement results. A plausible explanation can be derived from the melting points (T_melt_) and Tammann temperatures (T_Tam_) of the respective materials.

The Tammann temperature, defined empirically as by *T*_Tam_ = 0.67 · *T*_melt_ (in K), serves as an indicator of atomic mobility below the melting point and is often used to estimate the onset of significant diffusion processes. The melting points of Nb_2_O_5_, Ta_2_O_5,_ and SiO_2_ are 1512 °C, 1872 °C and 1710 °C. The Tammann temperature for these substances is reached at 905 °C for Nb_2_O_5_, 1035 °C for SiO_2,_ and only at 1142 °C for Ta_2_O_5_. At calcination at 1100 °C, the Tamann temperature of SiO_2_ is reached, which results in the sintering of the silica shells. In the case of Nb_2_O_5_, the Tamann temperature is also passed, which allows the core particles to grow in size. Conversely, for Ta_2_O_5_ the Tamann temperature is not reached, hindering the particles from sintering. This analysis provides a plausible explanation for the observed differences in particle growth behavior between Nb_2_O_5_ and Ta_2_O_5_ samples above 1000 °C. The higher Tammann temperature of Ta_2_O_5_ effectively inhibits significant particle growth, while Nb_2_O_5_ particles are more susceptible to sintering and growth under the same conditions.

## Conclusions

In this study, we have systematically explored the synthesis of niobium and tantalum oxide nanoparticles below 10 nm, achieving unprecedented control over their size and crystal structure, even at high-temperature conditions. For the first time, we successfully synthesized a series of niobium oxide polymorphs—NbO, NbO_2_, T-Nb_2_O_5_, M-Nb_2_O_5_, and H-Nb_2_O_5_—at the nanoscale while preserving their distinct structural and electronic properties. Our controlled synthesis approach, based on a single starting material and atmosphere-controlled thermal treatments, enables a comprehensive and systematic access to all major niobium oxide polymorphs at the nanoscale for the first time. Additionally, we synthesized crystalline tantalum oxide nanoparticles, which also demonstrated remarkable stability and controlled size. Our findings not only expand the repertoire of niobium and tantalum oxide polymorphs accessible at the size of 10 nm but also provide a robust framework for future investigations into the intrinsic properties of these materials. The preservation of nanoscale dimensions ensures that the unique physicochemical properties of these nanoparticles can be fully exploited, paving the way for innovative applications and deeper insights into their structure–property relationships.

## Supplementary Material



## Notes

Synthesis in literature and additional experimental details, Rietveld refinement fittings and additional XRD measurements, EDX elemental maps, and additional STEM images and Tauc plots for determination of optical bandgaps (PDF)
