# Supplementary material for: Size-Controlled High-Temperature Synthesis of Crystalline Niobium and Tantalum Oxide Nanoparticles: Exploring Structural Variations at Nanoscale
Source: Inorg Chem. 2025 Nov 17;64(47):23319–28. doi: 10.1021/acs.inorgchem.5c04574 (PMC12673515; doi:10.1021/acs.inorgchem.5c04574)
Supplement: Supplementary file 1 [file ic5c04574_si_001.pdf]

Supporting Information

Size-controlled high-temperature synthesis of crystalline niobium and tantalum oxide nanoparticles: exploring structural variations at nanoscale

Philipp Pfeifer,[*a] Souriddha Sanyal,[a] Marko Malinovic,[a] Andreas Göpfert,[b] Andreas Hutzler,[b] Huize Wang,[b] Marc Ledendecker[*a,b]

a) Technical University of Munich, Campus Straubing for Biotechnology and Sustainability, Sustainable Energy Materials, Schulgasse 22, 94315 Straubing, Germany
b) Helmholtz Institute Erlangen-Nürnberg for Renewable Energy, Forschungszentrum Jülich GmbH, Cauerstraße 1, 91058 Erlangen, Germany
*Philipp Pfeifer: philipp.pfeifer@tum.de, Marc Ledendecker: marc.ledendecker@tum.de

A – Synthesis in literature and additional experimental data

Table S1: Overview of amorphous and crystalline nanoparticles of niobium and tantalum oxides in literature and in this work, showing the crystalline phase of the material, particle size, and synthesis method. Subsequent heat treatments are signaled by the respective temperature.

Phase	Particle Size / nm	Synthesis Method + Calcination	Source
Amorphous NbO_x	9.4	RME + 600 °C	This work
Amorphous NbO_x	20, 30, 40	Sol-gel	(*1*)
Amorphous NbO_x	27	Sputtering + 200-450 °C	(*2*)
Amorphous NbO_x	4.5	Sol-gel	(*3*)
Amorphous NbO_x	40-100	Sol-gel + 400 °C	(*3*)
Amorphous NbO_x	25	Hydrolysis	(*4*)
Crystalline but not specified	1.3*-1.7*	Solvothermal @ 300 °C	(*5*)
TT-Nb_2O_5	18-35	Solvothermal	(*6, 7*)
TT-Nb_2O_5	27	Sputtering + 450-600 °C	(*2*)
T-Nb_2O_5	9.3	RME + 800 °C	This work
T-Nb_2O_5	30-40	Hydrothermal	(*8*)
T-Nb_2O_5	<100 (tablets)	Hydrolysis + 600 °C	(*9*)
T-Nb_2O_5	20-40	Hydrothermal + 700 °C	(*10*)
T-Nb_2O_5	27-45 (agglomerates)	Sputtering + 600-800 °C	(*2*)
T-Nb_2O_5	71.4	Pechini method + 750 °C	(*11*)
T-Nb_2O_5	733.3	Sol-gel + 750 °C	(*11*)
M-Nb_2O_5	9.7	RME + 1000 °C	This work
M-Nb_2O_5	1-20	Thermal evaporation	(*12, 13*)
H-Nb_2O_5	20	Hydrolysis + 500 °C	(*14*)
H-Nb_2O_5	1.4*	Solvothermal @ 160 °C	(*15*)
H-Nb_2O_5	2.0*	Solvothermal @ 200 °C	(*15*)
H-Nb_2O_5	2.6*	Solvothermal @ 300 °C	(*15*)
H-Nb_2O_5	35	Co-precipitation	(*16*)
Nb@NbO_x	25	Sputtering	(*2*)
NbO	10	RME + reduction	This work
NbO	20	Ball milling	(*17*)
NbO	200	Commercial + 300 °C	(*18*)
NbO_2	10	RME + reduction	This work
NbO_2	20-160	Reduction in carbon foam	(*19*)
NbO_2	>100 (sintered)	Reduction of nanorod precursor	(*20*)
NbO_2	20 (sintered)	La reduced Nb_2O_5	(*21*)
Amorphous TaO_x	11.6	RME + 800 °C	This work
Amorphous TaO_x	15-20	Hydrolysis	(*22*)
Amorphous TaO_x	6, 9, 13, 15	RME	(*23*)
Amorphous TaO_x	5.7	RME	(*24*)
Amorphous TaO_x	20	Sol-gel	(*25*)
Amorphous TaO_x	30	Solvothermal	(*25*)
Amorphous TaO_x	39.6	Direct evaporation	(*26*)
δ-Ta_2O_5	10.1	RME + 900 °C	This work
δ-Ta_2O_5	20-30	Sol-gel + 700 °C	(*27*)
δ-TaO (Ta core)	10	Hydrogen arc plasma	(*28*)
δ-Ta_2O_5	>100 (sintered)	Sol-gel + 800 °C	(*25*)
δ-Ta_2O_5	>20 (sintered)	Solvothermal + 800 °C	(*25*)
δ-Ta_2O_5	40	Anodic arc in water	(*29*)
δ-Ta_2O_5	20-30	Co-precipitation + Hydrothermal	(*30*)
δ-Ta_2O_5	40	Solvothermal + 800 °C	(*31, 32*)
$HTaO_3$	12-22	Sol-gel + Hydrothermal	(*33*)

* crystallite size from refinement

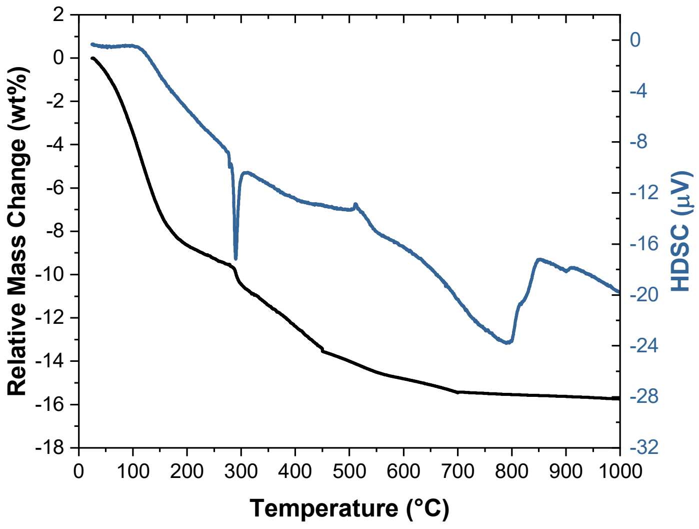

Figure S1: Weight loss curve (black) and heat flow between sample and blank (blue) determined by thermogravimetric analysis and differential scanning calorimetry of $NbO_x@SiO_2$ nanoparticles. Exothermic events are in the negative direction.

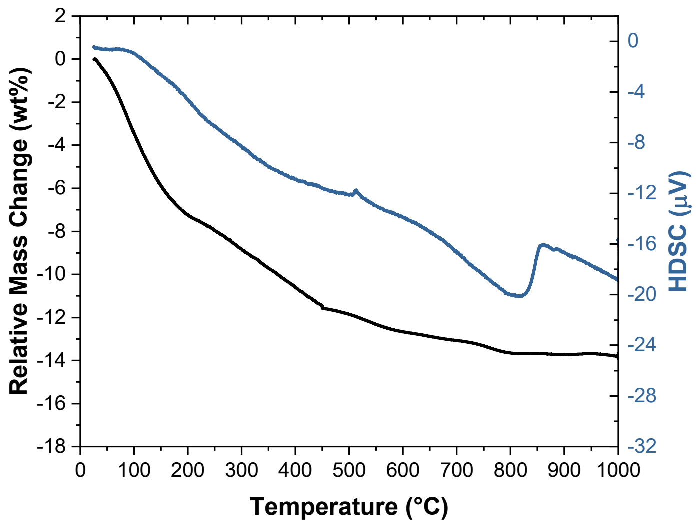

Figure S2: Weight loss curve (black) and heat flow between sample and blank (blue) determined by thermogravimetric analysis and differential scanning calorimetry of $TaO_x@SiO_2$ nanoparticles. Exothermic events are in the negative direction.

Table S2: Mass fractions of Si and Nb atoms and standard deviation as errors determined by XRF measurement of selected samples.

Sample	Si (wt%)	Nb (wt%)
$NbO_x@SiO_2$-400°C	86.43±3.20	13.57±3.20
$NbO_x@SiO_2$-600°C	85.32±3.04	14.68±3.04
$NbO_x@SiO_2$-800°C	82.62±5.98	17.38±5.98
$NbO_x@SiO_2$-900°C	80.02±6.42	19.98±6.42
$NbO_x@SiO_2$-1000°C	86.54±3.39	13.46±3.39
$NbO_x@SiO_2$-5% H_2	84.81±5.18	15.19±5.18
$NbO_x@SiO_2$-20% H_2	83.83±3.89	16.17±3.89
$NbO_x@SiO_2$-50% H_2	71.68±12.79	28.32±12.79
$NbO_x@SiO_2$-80% H_2	80.15±5.30	19.85±5.30

B – Rietveld Refinement and additional XRD measurements

All Rietveld refinements were carried out using the *Profex* software (*34*) that is based on the *BGMN* program. The structure files for the Rietveld refinement were derived from different sources, namely the Crystallographic Open Database (COD), the Inorganic Crystal Structure Database (ICSD) and in one case the Joint Committee on Powder Diffraction (JCPDS). An overview of the considered crystallographic data, as well as the databases and source, is given in the following Table S3. For all Rietveld refinements, the crystallite size distribution k1, the peak broadening parameter B1, as well as the lattice parameters were refined. The atomic coordinates were not refined. For the background, a Lagrangian polynomial was used. The silica peak was modelled using the amorphous function in the *Profex* software. The crystallite sizes are calculated in the *BGMN* environment based on the peak broadening described by the refined parameters k1 and B1. The crystallinity is estimated by applying the XRD integration method (*35*) (see also www.mcl.mse.utah.edu/xrd-crystallinity-by-integration/ for a tutorial) on the Rietveld refinement results. The area under all crystalline phases is integrated and divided by the integrated area of the whole diffraction pattern.

Table S3: Crystal phases, lattice parameters, database entries, and sources used for Rietveld refinement.

Phase	Space group	Lattice parameters (Å)			COD	JCPDS	ICSD	Source
		a	b	c				
T-Nb_2O_5	Pbam	6.17500	29.17500	3.93000	2106534		1591813	(*36*)
M-Nb_2O_5	I4/mmm	20.44000		3.83200	1528723		17027	(*37*)
H-Nb_2O_5	P2/m	21.19000	3.82900	19.39500		00-068-0149	29	(*38*)
NbO_2	$I4_1/a$	4.84630		3.03150	1548821		75198	(*39*)
NbO	$Pm\bar{3}m$	4.20200			1010410		27574	(*40*)
δ-Ta_2O_5	Pmm2	43.9969	3.894	6.209	1540126		66366	(*41*)

In the following, the results of the Rietveld refinements are shown in tabular form and plotted as background-corrected and deconvoluted diffraction patterns.

Table S4: Rietveld refinement results of temperature variation study on $NbO_x@SiO_2$. The table shows the identified phase, their mass fraction, lattice parameters, crystallite size, estimated crystallinity, goodness of fit (GoF), and weighted profile residual (R_{wp}) as measures for the quality of the fit.

Sample	Phase	Mass fraction (%)	Lattice parameter (Å) a	b	c	Size (nm)	Crystallinity (%)	GoF (≤2)	R_{wp} (%)
**400°C**	Amorphous	-	-	-	-	-	-	-	-
**600°C**	Amorphous	-	-	-	-	-	-	-	-
**800°C**	T-Nb_2O_5	100	6.1629(0)	28.883(2)	3.945(0)	3.76	6.29	0.84	2.97
**900°C**	T-Nb_2O_5	100	6.1133(0)	28.883(1)	3.9(6)	4.07	7.59	0.90	3.31
**1000°C**	T-Nb_2O_5	66.6	6.183(5)	29(1)	3.9504(7)	4.6	7.20	1.43	2.10
	M-Nb_2O_5	33.4	20.316(4)	-	3.8326(7)	23.7			
**1100°C**	M-Nb_2O_5	44.4	20.3466(3)	-	3.83(3)	40.8	18.47	1.48	2.20
	H-Nb_2O_5	53.8	28.7951(3)	3.83804(0)	17.655(0)	2.0			
	Silica	1.8	-		-	-			

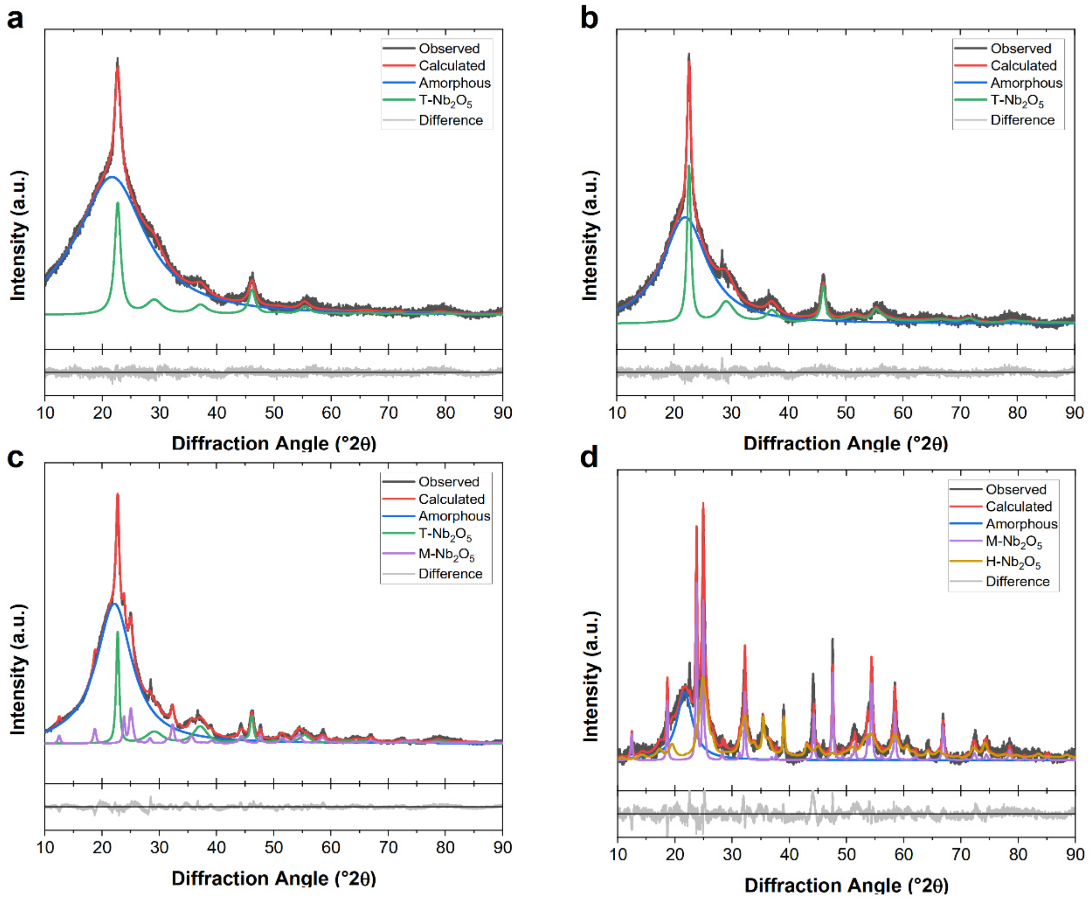

Figure S3: Background-corrected XRD patterns of $NbO_x@SiO_2$-800 °C (a), $NbO_x@SiO_2$-900 °C (b), $NbO_x@SiO_2$-1000 °C (c), and $NbO_x@SiO_2$-1100 °C (d). Shown are the observed data (black), the calculated Rietveld refinement fit (red), and the difference curve (gray). The calculated Rietveld refinement is further deconvoluted into individual crystalline and amorphous phase contributions.

Table S5: Rietveld refinement results of reductive atmosphere variation study on NbO_x@SiO_2. The table shows the identified phase, their mass fraction, lattice parameters, crystallite size, estimated crystallinity, goodness of fit (GoF), and weighted profile residual (R_{wp}) as measures for the quality of the fit.

Sample	Phase	Mass fraction (%)	Lattice parameter (Å)			Size (nm)	Crystallinity (%)	GoF (≤2)	R_{wp} (%)
			a	b	c				
**0% H_2**	NbO_2	100	4.8388(6)	-	3.0052(4)	8.0	12.39	1.12	3.85
	NbO	0	-	-	-	-			
**5% H_2**	NbO_2	74.6	4.8369(8)	-	3.0095(5)	7.5	16.21	0.96	3.59
	NbO	25.4	4.4067(0)	-	-	6.9			
**30% H_2**	NbO_2	33.0	4.8311(2)	-	3.0618(0)	11.9	16.63	1.05	3.61
	NbO	67.0	4.4097(6)	-	-	8.1			
**50% H_2**	NbO_2	0	-	-	-	-	17.65	1.07	3.77
	NbO	100	4.4111(4)	-	-	7.1			
**80 % H_2**	NbO_2	5.2	4.8598(0)	-	3.0618(0)	10.1	17.61	1.05	3.73
	NbO	94.8	4.4140(6)	-	-	6.8			
**80% H_2 reproduced***	NbO_2	0	-	-	-	-	9.79	1.02	3.32
	NbO	100	4.4046(6)	-	-	7.3			

* Sample NbO_x@SiO_2-80% H_2 was reproduced as it showed reflections of NbO_2 that accounted for 5.2 wt% in the sample. The reproduced sample is from a different synthesis batch and shows a higher SiO_2 content, which is evident in the lower crystallinity and a relatively higher amorphous silica peak in the diffraction pattern compared to the original sample.

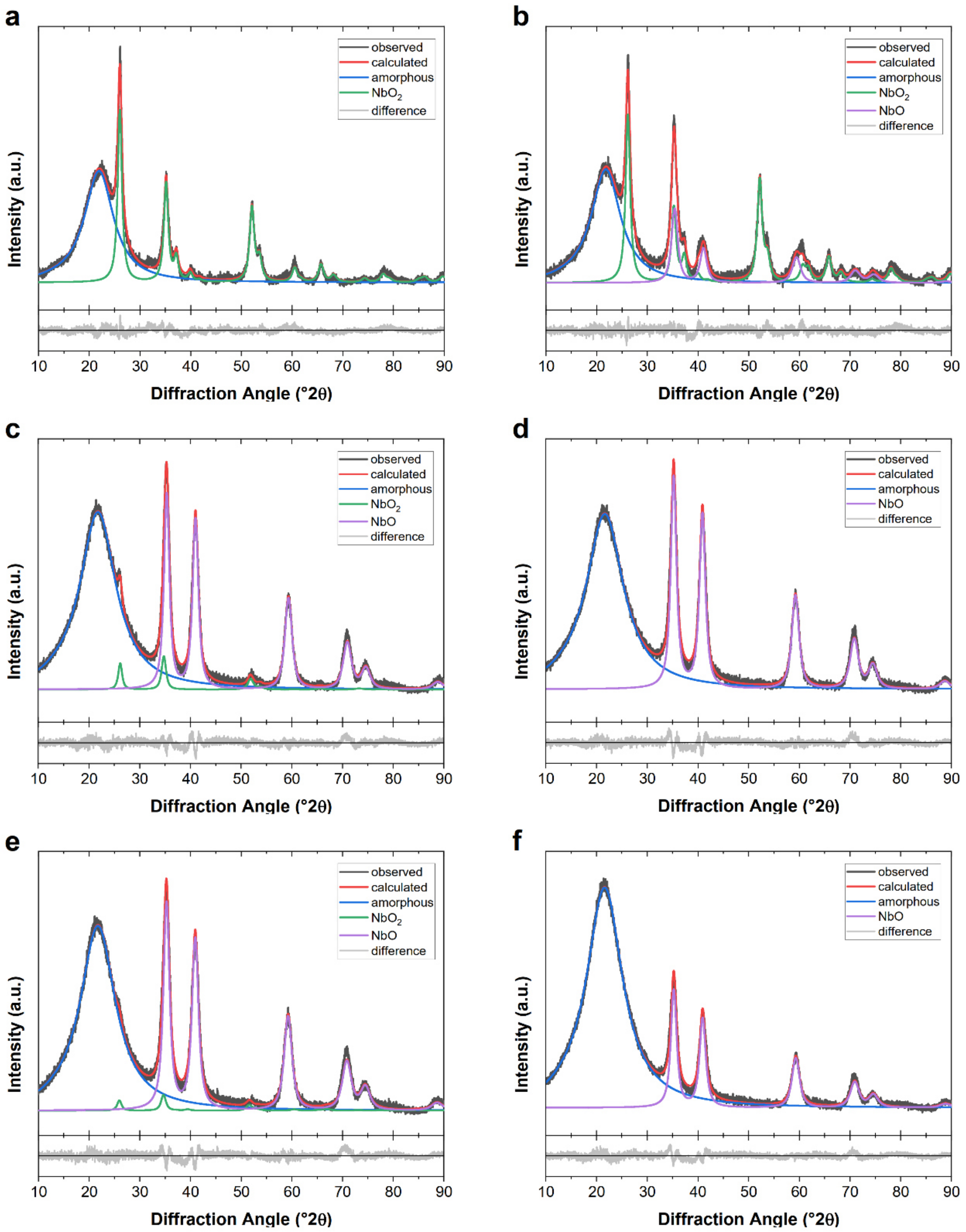

Figure S4: Background-corrected XRD patterns of $NbO_x@SiO_2$-0 % H_2 (a), $NbO_x@SiO_2$-5 % H_2 (b), $NbO_x@SiO_2$-30 % H_2 (c), $NbO_x@SiO_2$-50 % H_2 (d), $NbO_x@SiO_2$-80 % H_2 (e), $NbO_x@SiO_2$-80 % H_2 reproduced (f). Shown are the observed data (black), the calculated Rietveld refinement fit (red), and the difference curve (gray). The calculated Rietveld refinement is further deconvoluted into individual crystalline and amorphous phase contributions.

Table S6: Rietveld refinement results of temperature variation study in synthetic air on $TaO_x@SiO_2$. The table shows the identified phase, their mass fraction, lattice parameters, crystallite size, estimated crystallinity, goodness of fit (GoF), and weighted profile residual (R_{wp}) as measures for the quality of the fit.

Sample	Phase	Mass fraction (%)	Lattice parameter (Å)			Size (nm)	Crystallinity (%)	GoF (≤2)	R_{wp} (%)
			a	b	c				
**800°C**	Amorphous	-	-	-	-	-	-	-	-
**900°C**	Ta_2O_5	100	43.5569(0)	3.870(3)	6.181(7)	3.77	10.32	0.73	4.08
**1000°C**	Ta_2O_5	100	43.5569(0)	3.886(2)	6.186(5)	4.90	11.44	0.77	4.51
**1100°C**	Ta_2O_5	100	43.5569(0)	3.904(2)	6.1469(0)	5.63	13.14	0.86	5.30

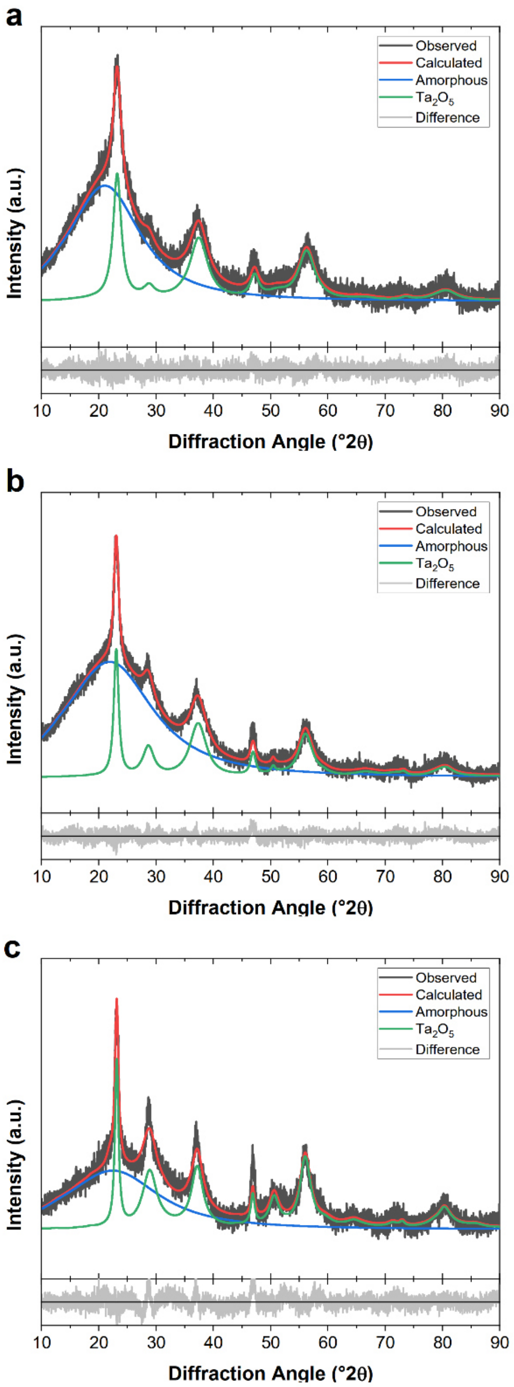

Figure S5: Background-corrected XRD patterns of Ta_2O_5@SiO_2 900 °C (a), Ta_2O_5@SiO_2 1000 °C (b) and Ta_2O_5@SiO_2 1100 °C (c). Shown are the observed data (black), the calculated Rietveld refinement fit (red), and the difference curve (gray). The calculated Rietveld refinement is further deconvoluted into individual crystalline and amorphous phase contributions.

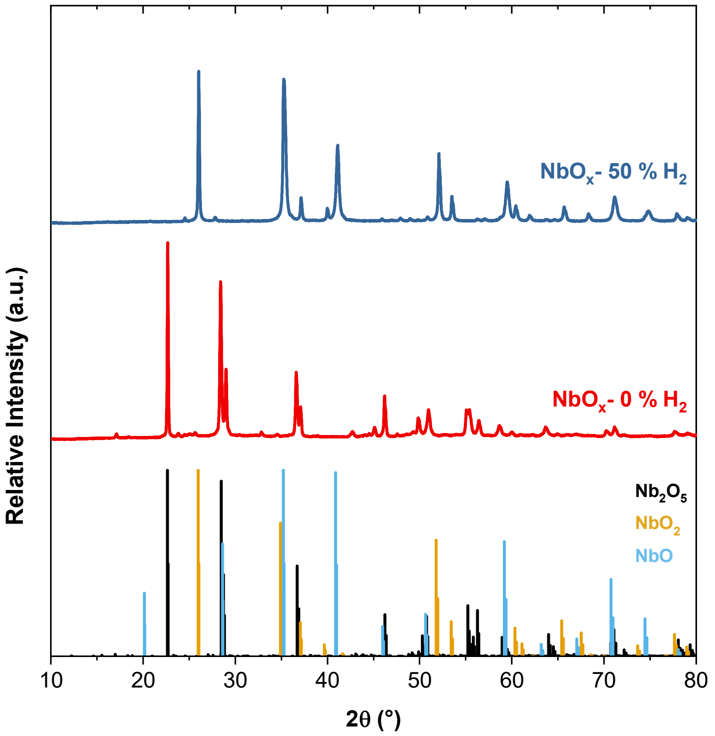

Figure S6: XRD diffraction patterns of NbO_x without silica shell and reference XRD data of Nb_2O_5, NbO_2, and NbO.

Table S7: Rietveld refinement results of control experiment of NbO_x without a silica shell in inert and reductive atmosphere. The table shows the identified phases, their mass fraction, lattice parameters, crystallite sizes, estimated crystallinity, goodness of fit (GoF), and weighted profile residual (R_{wp}) as measures for the quality of the fit.

Sample	Phase	Mass fraction (%)	Lattice parameter (Å)			Size (nm)	GoF (≤2)	R_{wp} (%)
			a	b	c			
**NbO_x- 0 % H_2**	T-Nb_2O_5	100	6.175	29.289	3.934	77.0	3.14	9.84
**NbO_x- 50 % H_2**	NbO_2	52.2	4.844	-	2.996	98.30	2.49	6.18
	NbO	47.8	4.395	-	-	39.87		

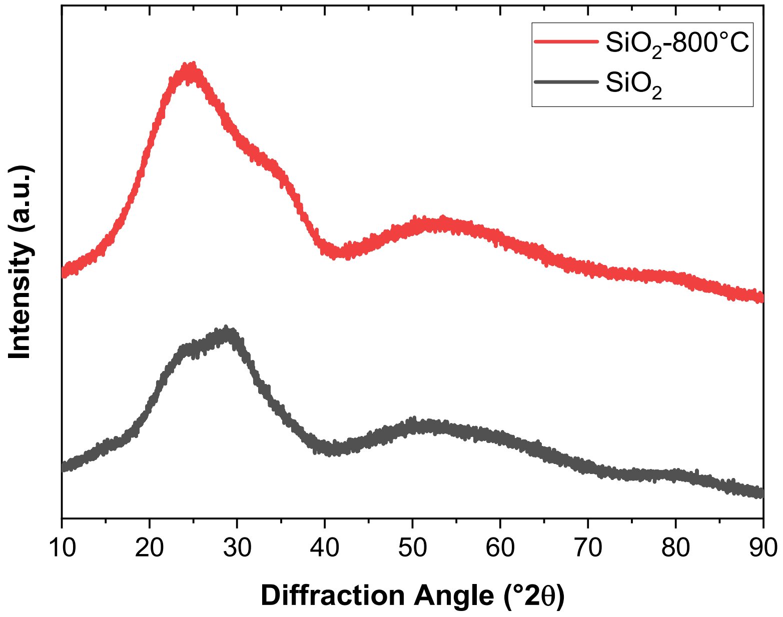

Figure S7: Diffraction patterns of SiO_2 before (black) and after calcination at 800°C (red).

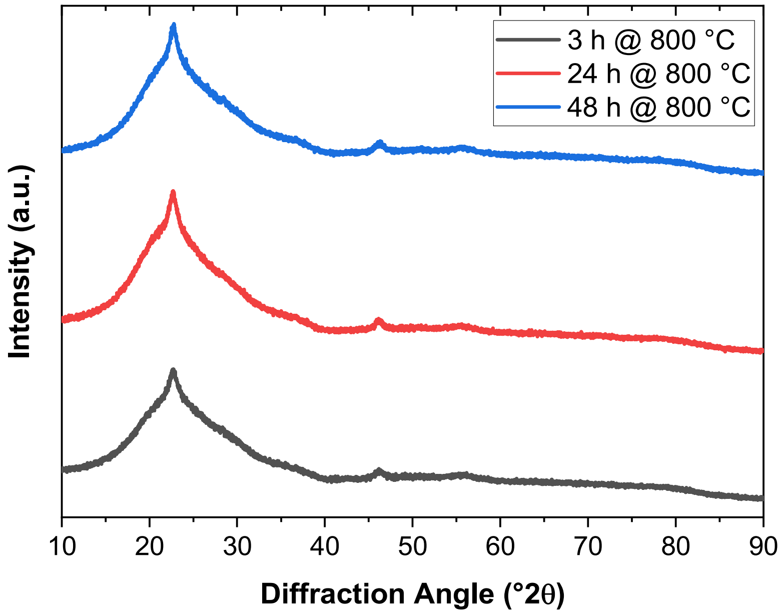

Figure S8: Diffraction patterns of NbO_x@SiO_2 calcinated at 800 °C for 3 h (black), 24 h (red) and 48 h (blue).

C – EDX elemental maps and additional STEM images

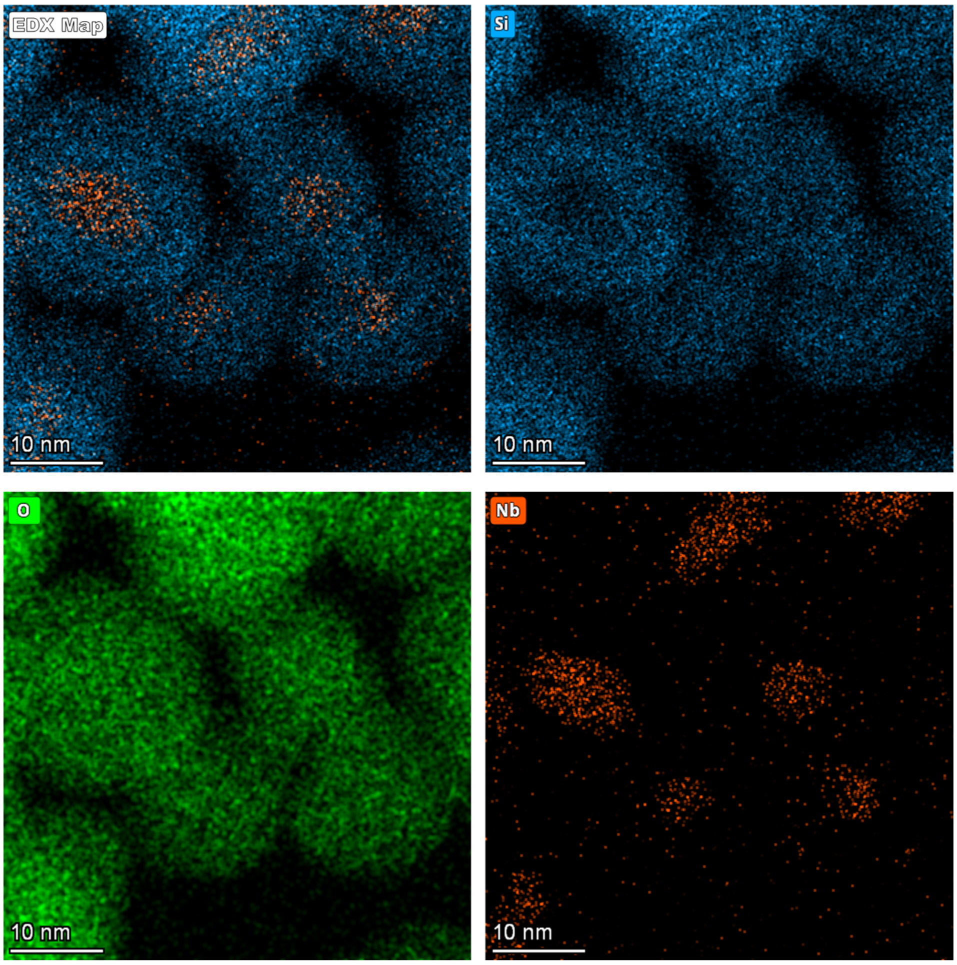

Figure S9: EDX elemental maps of $NbO_x@SiO_2$-900 °C showing the distribution of silicon (blue), oxygen (green), and niobium (orange).

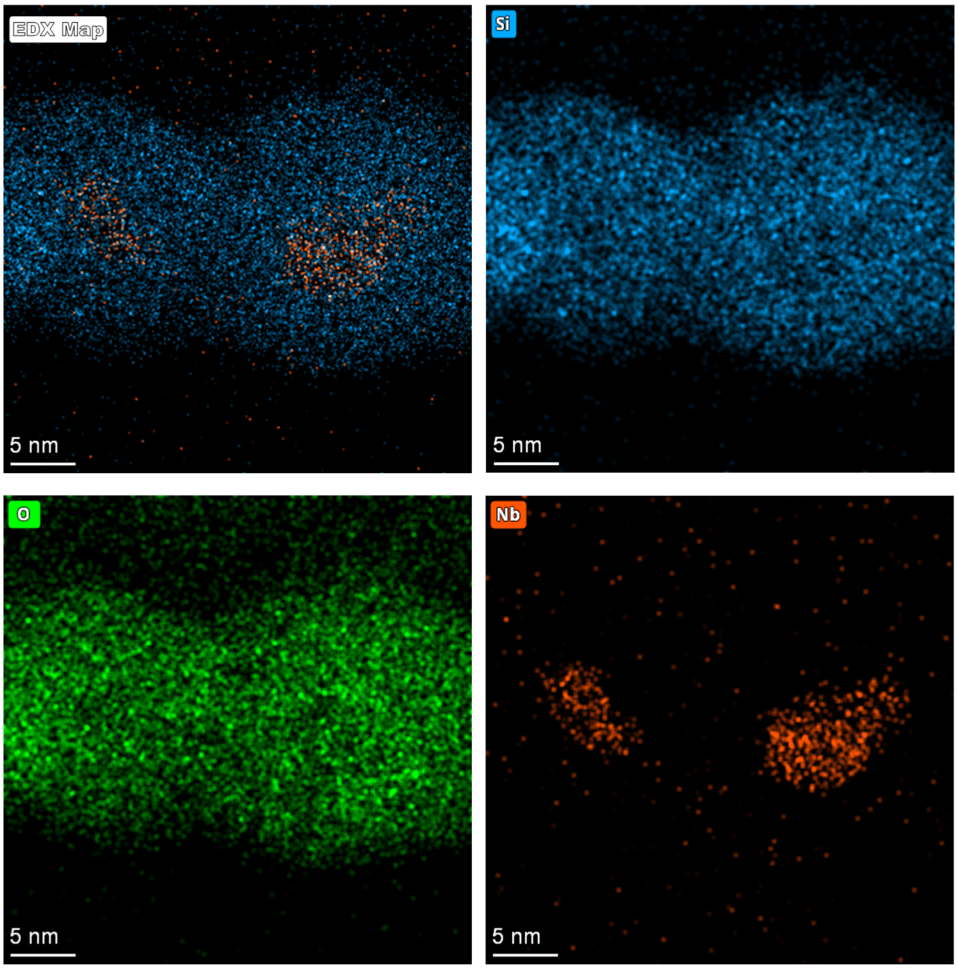

Figure S10: EDX elemental maps of $NbO_x@SiO_2$-50 % H_2 showing the distribution of silicon (blue), oxygen (green), and niobium (orange).

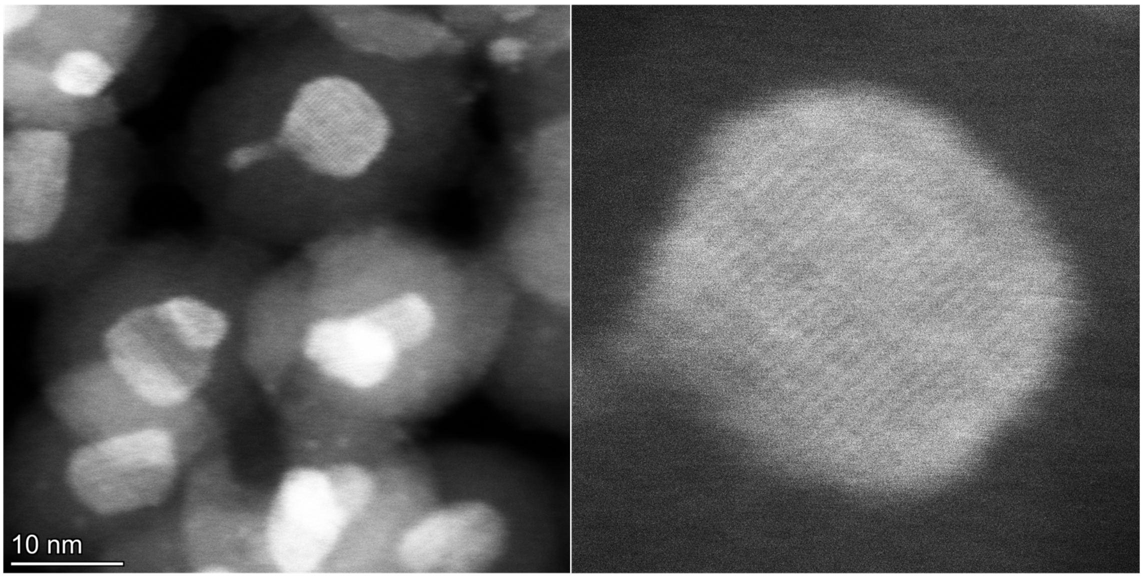

Figure S11: HAADF image of $NbO_x@SiO_2$-900 °C. The particle showing visible lattice fringes is enlarged in the right image.

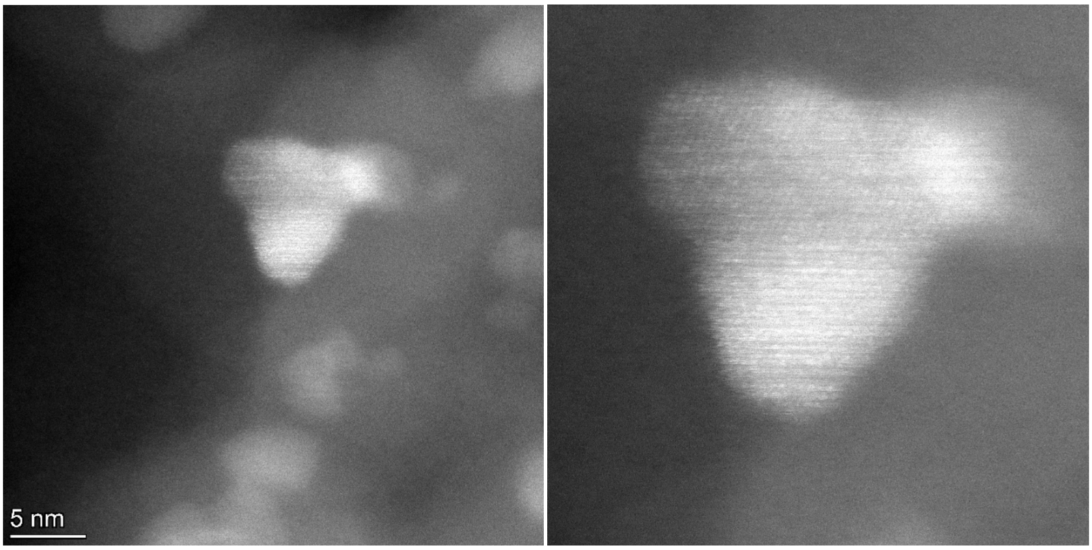

Figure S12: HAADF image of $NbO_x@SiO_2$-50 % H_2. The particle showing visible lattice fringes is enlarged in the right image.

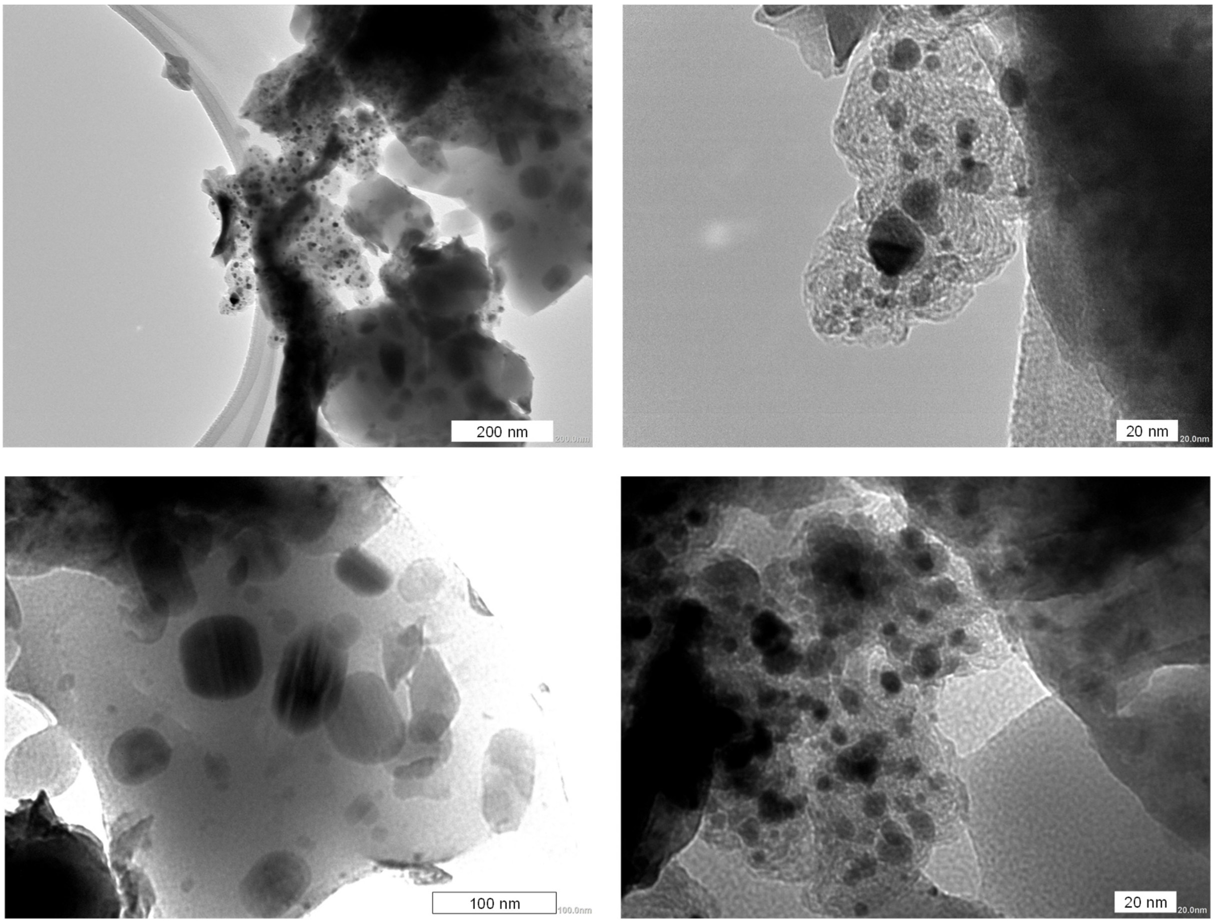

Figure S13: TEM images of $NbO_x@SiO_2$-1100 °C.

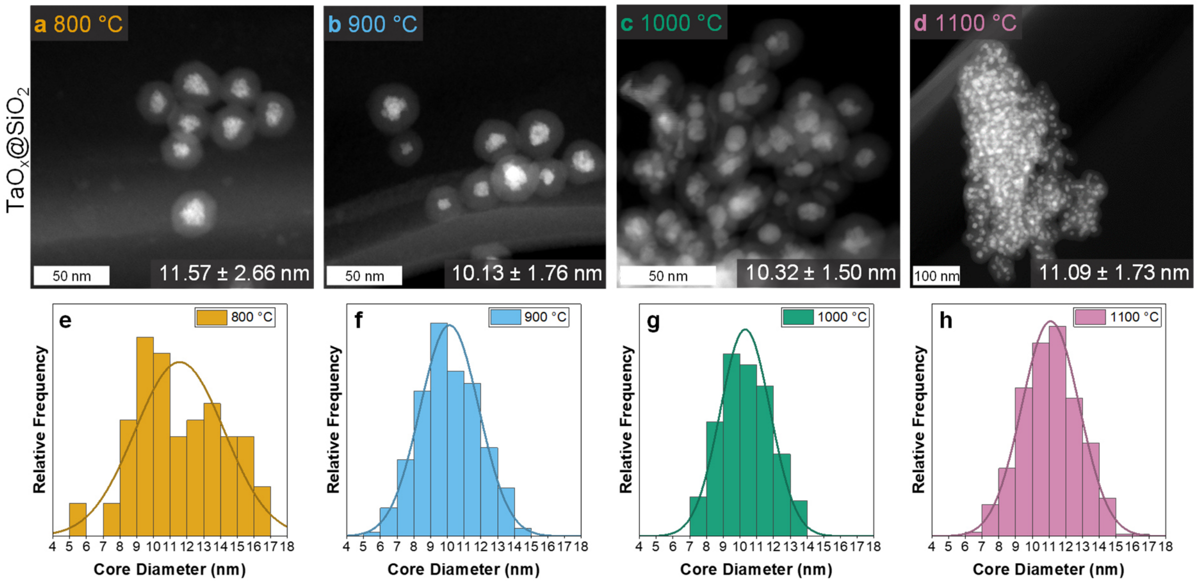

Figure S14: HAADF-STEM image of $TaO_x@SiO_2$ (a-d), and corresponding particle size distributions (e-h).

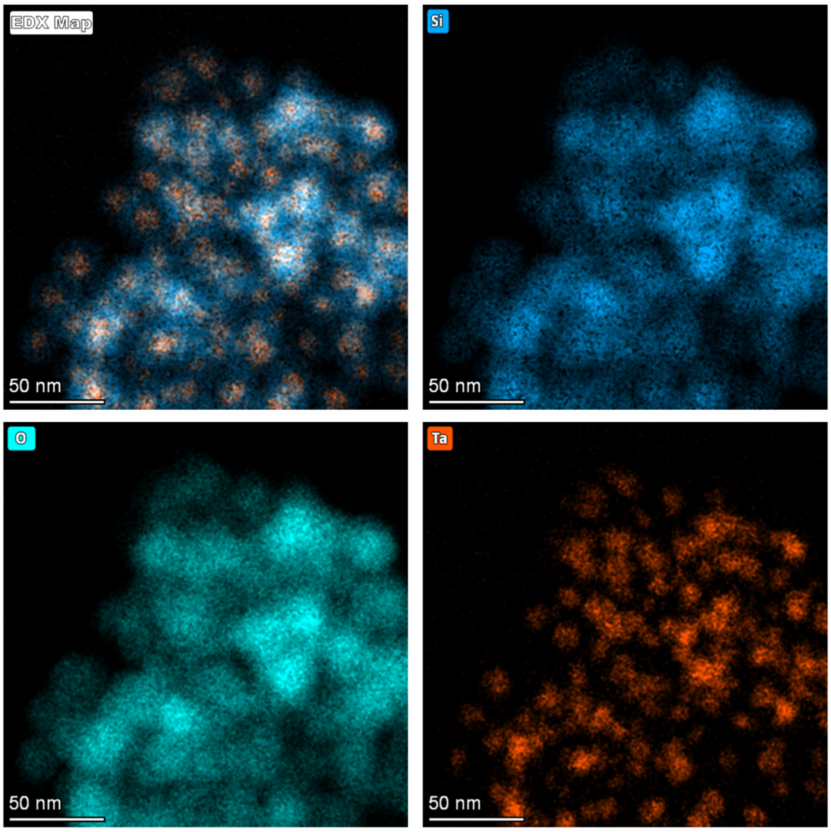

Figure S15: EDX elemental maps of $TaO_x@SiO_2$-1000 °C showing the distribution of silicon (blue), oxygen (turquoise), and tantalum (orange).

D – Tauc-plots for determination of optical bandgaps

For UV-vis measurements the measured reflectance of the sample R_{sample} and the $BaSO_4$ standard $R_{standard}$ were used to calculate the absorbance following the Kubelka-Munk equation (1):

$$F(R_\infty) = \frac{(1 - R_\infty)^2}{2 \cdot R_\infty} \quad (1)$$

With R_∞ (2):

$$R_\infty = \frac{R_{sample}}{R_{standard}} \quad (2)$$

For plotting, the Tauc-equation (3) is used with h being the Planck constant, ν the light frequency, γ a factor depending on the nature of the band gap, B is a constant, and E_g being the bandgap energy.

$$(F(R_\infty) \cdot h\nu)^{1/\gamma} = B(h\nu - E_g) \quad (3)$$

Nb_2O_5 polymorphs possess a direct bandgap. This means that with $\gamma = \frac{1}{2}$ the equation transforms to equation (4):

$$(F(R_\infty) \cdot h\nu)^2 = B(h\nu - E_g) \quad (4)$$

The left side of the equation becomes zero when $h\nu = E_g$ therefore, the bandgap can be read on the intersection of the linear part of the curve and the abscissa.

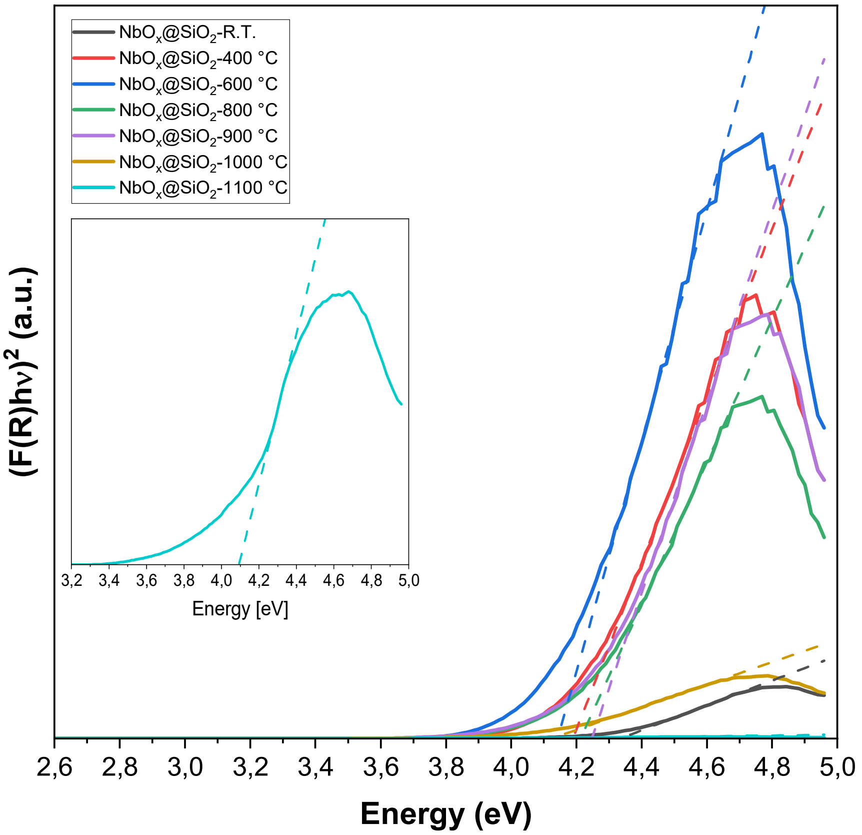

Figure S16: Tauc plots of NbOx@SiO_2 nanoparticles calcinated in synthetic air at different temperatures. The inset shows the enlarged NbO_x@SiO_2 -1100 °C.

References

1. Falk, G.; Borlaf, M.; Bendo, T.; Novaes de Oliveira, A. P.; Rodrigues Neto, J. B.; Moreno, R. Colloidal Sol–Gel Synthesis and Photocatalytic Activity of Nanoparticulate Nb 2 O 5 Sols. *J. Am. Ceram. Soc.* **2016,** *99* (6), 1968–1973. DOI: 10.1111/jace.14217.

2. Košutová, T.; Horák, L.; Pleskunov, P.; Hanuš, J.; Nikitin, D.; Kúš, P.; Cieslar, M.; Gordeev, I.; Burazer, S.; Choukourov, A.; Dopita, M. Thermally-driven morphogenesis of niobium nanoparticles as witnessed by in-situ x-ray scattering. *Materials Chemistry and Physics* **2022,** *277*, 125466. DOI: 10.1016/j.matchemphys.2021.125466.

3. Brayner, R.; Bozon-Verduraz, F. Niobium pentoxide prepared by soft chemical routes: morphology, structure, defects and quantum size effect. *Phys. Chem. Chem. Phys.* **2003,** *5* (7), 1457–1466. DOI: 10.1039/B210055J.

4. Onur, E.; Lee, J.; Aymerich-Armengol, R.; Lim, J.; Dai, Y.; Tüysüz, H.; Scheu, C.; Weidenthaler, C. Exploring the Effects of the Photochromic Response and Crystallization on the Local Structure of Noncrystalline Niobium Oxide. *ACS Applied Materials & Interfaces* **2024,** *16* (19), 25136–25147. DOI: 10.1021/acsami.4c04038.

5. Kjær, E. T. S.; Aalling-Frederiksen, O.; Yang, L.; Thomas, N. K.; Juelsholt, M.; Billinge, S. J. L.; Jensen, K. M. Ø. In Situ Studies of the Formation of Tungsten and Niobium Oxide Nanoparticles: Towards Automated Analysis of Reaction Pathways from PDF Analysis using the Pearson Correlation Coefficient. *Chemistry Methods* **2022,** *2* (9), e202200034. DOI: 10.1002/cmtd.202200034.

6. Buha, J.; Arčon, D.; Niederberger, M.; Djerdj, I. Solvothermal and surfactant-free synthesis of crystalline Nb(2)O(5), Ta(2)O(5), HfO(2), and Co-doped HfO(2) nanoparticles. *Phys. Chem. Chem. Phys.* **2010,** *12* (47), 15537–15543. DOI: 10.1039/c0cp01298j.

7. Rani, R. A.; Zoolfakar, A. S.; O'Mullane, A. P.; Austin, M. W.; Kalantar-Zadeh, K. Thin films and nanostructures of niobium pentoxide: fundamental properties, synthesis methods and applications. *J. Mater. Chem. A* **2014,** *2* (38), 15683–15703. DOI: 10.1039/C4TA02561J.

8. Lopes, O. F.; Paris, E. C.; Ribeiro, C. Synthesis of Nb_2O_5 nanoparticles through the oxidant peroxide method applied to organic pollutant photodegradation: A mechanistic study. *Applied Catalysis B: Environmental* **2014,** *144*, 800–808. DOI: 10.1016/j.apcatb.2013.08.031.

9. Gómez, C.; Rodríguez-Páez, J. The effect of the synthesis conditions on structure and photocatalytic activity of Nb2O5 nanostructures. *PAC* **2018,** *12* (3), 218–229. DOI: 10.2298/PAC1803218G.

10. Kong, L.; Zhang, C.; Zhang, S.; Wang, J.; Cai, R.; Lv, C.; Qiao, W.; Ling, L.; Long, D. High-power and high-energy asymmetric supercapacitors based on Li + -intercalation into a T-Nb 2 O 5 /graphene pseudocapacitive electrode. *J. Mater. Chem. A* **2014,** *2* (42), 17962–17970. DOI: 10.1039/C4TA03604B.

11. Joya, M.; Barba Ortega, J.; Raba Paez, A.; Da Silva Filho, J.; Cavalcante Freire, P. Synthesis and Characterization of Nano-Particles of Niobium Pentoxide with Orthorhombic Symmetry. *Metals* **2017,** *7* (4), 142. DOI: 10.3390/met7040142.

12. Dhawan, S.; Dhawan, T.; Vedeshwar, A. G. Growth of Nb2O5 quantum dots by physical vapor deposition. *Materials Letters* **2014,** *126*, 32–35. DOI: 10.1016/j.matlet.2014.03.107.

13. Nico, C.; Monteiro, T.; Graça, M. Niobium oxides and niobates physical properties: Review and prospects. *Progress in Materials Science* **2016,** *80*, 1–37. DOI: 10.1016/j.pmatsci.2016.02.001 (accessed 2024-01-19).

14. Athar, T.; Hashmi, A.; Al-Hajry, A.; Ansari, Z. A.; Ansari, S. G. One-pot synthesis and characterization of Nb_2O_5 nanopowder. *Journal of nanoscience and nanotechnology* **2012,** *12* (10), 7922–7926. DOI: 10.1166/jnn.2012.6645 (accessed 2024-08-12).

15. Aalling-Frederiksen, O.; Juelsholt, M.; Anker, A. S.; Jensen, K. M. Ø. Formation and growth mechanism for niobium oxide nanoparticles: atomistic insight from in situ X-ray total scattering. *Nanoscale* **2021,** *13* (17), 8087–8097. DOI: 10.1039/d0nr08299f.

16. Kumar, K. Y.; Prashanth, M. K.; Shanavaz, H.; Parashuram, L.; Alharti, F. A.; Jeon, B.-H.; Raghu, M. S. Green and facile synthesis of strontium doped Nb2O5/RGO photocatalyst: Efficacy towards H2 evolution, benzophenone-3 degradation and Cr(VI) reduction. *Catalysis Communications* **2023,** *173*, 106560. DOI: 10.1016/j.catcom.2022.106560.

17. Valeeva, A. A.; Schroettner, H.; Rempel, A. A. NbO disintegration by surfactant-assisted high-energy ball milling. *Inorg Mater* **2014,** *50* (4), 398–403. DOI: 10.1134/S0020168514040177.

18. Nico, C.; Soares, M. R. N.; Rodrigues, J.; Matos, M.; Monteiro, R.; Graça, M. P. F.; Valente, M. A.; Costa, F. M.; Monteiro, T. Sintered NbO Powders for Electronic Device Applications. *J. Phys. Chem. C* **2011,** *115* (11), 4879–4886. DOI: 10.1021/jp110672u.

19. Asfaw, H. D.; Tai, C.-W.; Nyholm, L.; Edström, K. Over-Stoichiometric NbO_2 Nanoparticles for a High Energy and Power Density Lithium Microbattery. *ChemNanoMat* **2017,** *3* (9), 646–655. DOI: 10.1002/cnma.201700141.

20. Huang, L.; Wu, J.; Han, P.; Al-Enizi, A. M.; Almutairi, T. M.; Zhang, L.; Zheng, G. NbO_2 Electrocatalyst Toward 32% Faradaic Efficiency for N_2 Fixation. *Small Methods* **2019,** *3* (6), 1800386. DOI: 10.1002/smtd.201800386.

21. Huang, C.; Dong, W.; Dong, C.; Wang, X.; Jia, B.; Huang, F. Niobium dioxide prepared by a novel La-reduced route as a promising catalyst support for Pd towards the oxygen reduction reaction. *Dalton Trans.* **2020,** *49* (5), 1398–1402. DOI: 10.1039/C9DT04570H.

22. Onur Şahin, E.; Tüysüz, H.; Chan, C. K.; Moon, G.-H.; Dai, Y.; Schmidt, W.; Lim, J.; Scheu, C.; Weidenthaler, C. In situ total scattering experiments of nucleation and crystallisation of tantalum-based oxides: from highly dilute solutions via cluster formation to nanoparticles. *Nanoscale* **2021,** *13* (1), 150–162. DOI: 10.1039/D0NR07871A.

23. Oh, M. H.; Lee, N.; Kim, H.; Park, S. P.; Piao, Y.; Lee, J.; Jun, S. W.; Moon, W. K.; Choi, S. H.; Hyeon, T. Large-scale synthesis of bioinert tantalum oxide nanoparticles for X-ray computed tomography imaging and bimodal image-guided sentinel lymph node mapping. *Journal of the American Chemical Society* **2011,** *133* (14), 5508–5515. DOI: 10.1021/ja200120k.

24. Zhang, Y.; Zabinyakov, N.; Majonis, D.; Bouzekri, A.; Ornatsky, O.; Baranov, V.; Winnik, M. A. Tantalum Oxide Nanoparticle-Based Mass Tag for Mass Cytometry. *Analytical chemistry* **2020,** *92* (8), 5741–5749. DOI: 10.1021/acs.analchem.9b04970.

25. Koshevaya, E. D.; Khramov, E. V.; Svetogorov, R. D.; Krasnov, A. G.; Martakov, I. S.; Shishkin, I. I.; Krivoshapkina, E. F.; Krivoshapkin, P. V. Stokes and Anti-Stokes Luminescent Rare-Earth-Doped Tantalum Oxide Nanoparticles. *Inorganic chemistry* **2023,** *62* (26), 10369–10381. DOI: 10.1021/acs.inorgchem.3c01231.

26. Zhang, N.; Li, L.; Li, G. Nanosized amorphous tantalum oxide: a highly efficient photocatalyst for hydrogen evolution. *Res Chem Intermed* **2017,** *43* (9), 5011–5024. DOI: 10.1007/s11164-017-3052-y.

27. Khanal, V.; Balayeva, N. O.; Günnemann, C.; Mamiyev, Z.; Dillert, R.; Bahnemann, D. W.; Subramanian, V. Photocatalytic NO_x removal using tantalum oxide nanoparticles: A benign pathway. *Applied Catalysis B: Environmental* **2021,** *291*, 119974. DOI: 10.1016/j.apcatb.2021.119974.

28. Wang, Y.; Cui, Z.; Zhang, Z. Synthesis and phase structure of tantalum nanoparticles. *Materials Letters* **2004,** *58* (24), 3017–3020. DOI: 10.1016/j.matlet.2004.05.031.

29. Delaportas, D.; Svarnas, P.; Alexandrou, I. Ta_2O_5 Crystalline Nanoparticle Synthesis by DC Anodic Arc in Water. *J. Electrochem. Soc.* **2010,** *157* (6), K138. DOI: 10.1149/1.3391610.

30. Li, Z.; Liu, J.; Li, J.; Shen, J. Template free synthesis of crystallized nanoporous F-Ta2O5 spheres for effective photocatalytic hydrogen production. *Nanoscale* **2012,** *4* (13), 3867–3870. DOI: 10.1039/c2nr30721a.

31. Manukumar, K. N.; Kishore, B.; Viswanatha, R.; Nagaraju, G. Ta_2O_5 nanoparticles as an anode material for lithium ion battery. *J Solid State Electrochem* **2020,** *24* (4), 1067–1074. DOI: 10.1007/s10008-020-04593-3.

32. Gurylev, V. A review on the development and advancement of Ta_2O_5 as a promising photocatalyst. *Materials Today Sustainability* **2022,** *18*, 100131. DOI: 10.1016/j.mtsust.2022.100131.

33. Gömpel, D.; Tahir, M. N.; Panthöfer, M.; Mugnaioli, E.; Brandscheid, R.; Kolb, U.; Tremel, W. Facile hydrothermal synthesis of crystalline Ta 2 O 5 nanorods, MTaO 3 (M = H, Na, K, Rb) nanoparticles, and their photocatalytic behaviour. *J. Mater. Chem. A* **2014,** *2* (21), 8033–8040. DOI: 10.1039/C4TA00183D.

34. Doebelin, N.; Kleeberg, R. Profex: a graphical user interface for the Rietveld refinement program BGMN. *Journal of applied crystallography* **2015,** *48* (Pt 5), 1573–1580. DOI: 10.1107/S1600576715014685.

35. Abdul Mannana; K R Kazmia; I H Khan; Muhammad Shafiq Khan. A Method for the Determination of Relative Crystallinity of Minerals by X-Ray Diffraction. *Pakistan Journal of Scientific & Industrial Research* **2006,** *49* (2), 72–76.

36. Kato, K.; Tamura, S. Die Kristallstruktur von T-Nb_2O_5. *Acta Crystallogr B Struct Sci* **1975,** *31* (3), 673–677. DOI: 10.1107/S0567740875003603.

37. Mertin, W.; Andersson, S.; Gruehn, R. Über die Kristallstruktur von M-Nb_2O_5. *Journal of Solid State Chemistry* **1970,** *1* (3-4), 419–424. DOI: 10.1016/0022-4596(70)90124-6.

38. Kato, K. Structure refinement of H-Nb_2O_5. *Acta Cryst B* **1976,** *32* (3), 764–767. DOI: 10.1107/S0567740876003944.

39. Bolzan, A. A.; Fong, C.; Kennedy, B. J.; Howard, C. J. A Powder Neutron Diffraction Study of Semiconducting and Metallic Niobium Dioxide. *Journal of Solid State Chemistry* **1994,** *113* (1), 9–14. DOI: 10.1006/jssc.1994.1334.

40. Brauer, G. Die Oxyde des Niobs. *Z. Anorg. Allg. Chem.* **1941,** *248* (1), 1–31. DOI: 10.1002/zaac.19412480101.

41. Hummel, H.-U.; Fackler, R.; Remmert, P. Tantaloxide durch Gasphasenhydrolyse, Druckhydrolyse und Transportreaktion aus 2H-TaS 2 : Synthesen von TT-Ta 2 O 5 und T-Ta 2 O 5 und Kristallstruktur von T-Ta 2 O 5. *Chem. Ber.* **1992,** *125* (3), 551–556. DOI: 10.1002/cber.19921250304.
